# AI-enhanced rapid diagnostic testing platform for mass opisthorchiasis screening

**DOI:** 10.1038/s41598-025-16893-7

**Published:** 2025-08-23

**Authors:** Prem Junsawang, Anchalee Techasen, Kannika Wiratchawa, Yupaporn Wanna, Phattharaphon Wongphutorn, Chanika Worasith, Paiboon Sithithaworn, Sahan Bulathwela, Thanapong Intharah

**Affiliations:** 1 Visual Intelligence Laboratory, Department of Statistics, Faculty of Science, Khon Kaen University, Khon Kaen, Thailand; 2 Faculty of Associated Medical Sciences, Khon Kaen University, Khon Kaen, Thailand; 3 Cholangiocarcinoma Research Institute, Khon Kaen University, Khon Kaen, Thailand; 4 Department of Parasitology, Faculty of Medicine, Khon Kaen University, Khon Kaen, Thailand; 5 Department of Adult Nursing, Faculty of Nursing, Khon Kaen University, Khon Kaen, Thailand; 6 Centre for Artificial Intelligence, Department of Computer Science, University College London, London, UK

**Keywords:** Information technology, Parasitic infection, Health policy, Computer science

## Abstract

Cholangiocarcinoma (CCA) is a prevalent malignancy in countries along Mekong basin, closely linked to chronic infections caused by *Opisthorchis viverrini* (OV). Early detection of OV-infected individuals holds significant promise for screening at-risk populations in endemic regions. Recent advancements in immunochromatographic methods have led to the development of a rapid diagnostic test (RDT) based on urinary antigens. However, the current interpretation relies on visual assessment of T-band color intensity, which can be subjective and prone to variability. Furthermore, aggregating data at the regional/country level demands data digitization, a time and resource intense task that introduces further errors. To address this limitation, we introduce the OV-RDT platform, a cloud-based system incorporating artificial intelligence (AI) designed to standardize the reading and interpretation of OV-RDT results while facilitating mass screening campaigns for opisthorchiasis. This cross-platform solution, available on Android and iOS devices, consists of three key components: a mobile application, an intelligent dashboard, and a cloud server cluster. The server cluster has two main components the data processing server and the AI server. The AI server operates two AI-based models systematically developed and validated for image quality assessment and T-band grading of OV-RDT test kit images. The data processing server periodically retrieves and processes data from the cloud database, enabling comprehensive daily visualization through the intelligent dashboard. Validation through extensive field testing was conducted specifically in the northeastern region of Thailand, where opisthorchiasis prevalence is among the highest globally, demonstrating remarkable effectiveness by processing over 100,000 samples. While our platform shows excellent performance in this endemic region, external validation in other geographical areas would be necessary to establish broader generalizability. The EfficientNet-B5-based deep learning model used in the platform exhibited impressive performance in both image quality assessment (98% accuracy) and infection grading classification (95% accuracy in detecting OV infection status). The platform’s user-friendly interface has achieved high satisfaction rates (4.41/5.00) among healthcare workers, while its intelligent dashboard offers real-time analytics and geospatial visualization capabilities. This integrated approach marks a significant advancement in mass screening for opisthorchiasis, potentially enhancing early detection rates and supporting more effective public health interventions in Southeast Asia and the Mekong Basin countries. This study addresses the critical need for mass screening in northeastern Thailand, where liver fluke infection rates are particularly severe; however, the platform’s performance in other regions requires future validation studies.

## Introduction

Cholangiocarcinoma (CCA) remains an extremely common malignancy in the northeastern region of Thailand and other countries located along the Mekong River. The disease is associated with a significant burden of morbidity and mortality. Chronic or recurrent infections with Opisthorchis viverrini are a significant risk factor for developing CCA^1,2^. The consumption of raw or partially cooked freshwater cyprinid fish by people in these regions serves as the primary route of O. viverrini infection During the early stages, CCA mostly exhibit either non-specific or asymptomatic symptoms, resulting in the emergence of a clinically significant picture only in the advanced stages of the disease^3^.

Khuntikeo et al.^4^ proposed a screening method that leads to early diagnosis of CCA. For that purpose, Khon Kaen University has established the “Cholangiocarcinoma Screening and Care Program” (CASCAP) in collaboration with the National Health Security Office (NHSO) and the Ministry of Public Health, Thailand. These organizations have implemented a policy to enhance the systematic diagnosis and treatment of people with CCA across the northeastern region of Thailand. The northeastern region of Thailand experiences particularly severe opisthorchiasis prevalence, with infection rates ranging from 20-70% in some provinces, making it one of the most affected areas globally and thus a critical target for screening interventions.

Currently, ultrasonography^5,6^ is a standard method for screening disorders of the biliary system. Chamadol et al.^7^ established a teleconsultation system called CASCAP MD KKU Solution, designed to assess a massive number of radiological images. Online consultation aids inexperienced medical workers in analyzing ultrasonographic findings. Although ultrasonography is highly accessible, general practitioners (GPs) or sonographers often require a second opinion from experienced radiologists to analyze. Intharah et al.^7^ recently developed a hybrid deep neural network model named biliary tract network (BiTNet) for the analysis of biliary tract abnormalities in ultrasound images. BiTNet is the first ultrasound image analysis model that can be applied for screening and assisting in diagnosing the human biliary tract from ultrasound images. The model aims to aid in two ways. First, pre-screening the ultrasound image as either normal or abnormal helps reduce the workload of radiologists. Second, using an assisting tool, GPs and radiologists can diagnose abnormalities from ultrasound images of the human biliary tract.

Besides ultrasound screening, detecting of individuals infected with OV has proven the potential to rapidly screen individuals at risk in places where the infection is prevalent. Worasith et al.^8^ developed a urinary antigen-based rapid diagnostic test (RDT) using immunochromatographic methodology to simplify diagnosis and field applications for surveillance and control of opisthorchiasis. The OV-RDT test has high potential as a new tool for screening and evaluating treatment outcomes in opisthorchiasis. The ease of use and cost effectiveness of OV-RDT may facilitate mass screening of opisthorchiasis. However, the interpretation of infection intensity relies on the visual assessment of color changes on the test line (T band), which could lead to inconsistencies among different raters, particularly during large-scale or point-of-care testing. To address this variability, utilizing technology such as Artificial Intelligence (AI) that could assist in result interpretation would be highly beneficial. The primary objective of this work is to create an AI-OVRDT system that could assist in reading and interpreting the results of OV-RDT and reporting on mass screening of opisthorchiasis.

In this work, we introduce the OV-RDT platform, a comprehensive solution combining artificial intelligence, cloud computing, and mobile technology to revolutionize mass opisthorchiasis screening campaigns. Our contributions include the development of a robust deep learning model based on EfficientNet-B5 architecture for standardized interpretation of OV-RDT results, achieving 95% accuracy in detecting OV infection status. Additionally, we implemented an AI-based quality control algorithm to ensure optimal rapid diagnostic test strip image capture and standardisation. We implement a scalable cloud-based infrastructure that enables efficient data collection, storage, and processing from multiple screening locations, supporting over 100,000 participants across northeastern Thailand. The platform features user-friendly mobile applications for both Android and iOS platforms, ensuring widespread accessibility for healthcare workers, with user satisfaction rates exceeding 4.40 out of 5.00 Additionally, we integrate an intelligent dashboard that provides real-time analytics and visualization of screening data, enabling evidence-based decision-making for public health interventions. Through extensive field testing in endemic areas of northeastern Thailand and validation with healthcare professionals, we demonstrate the platform’s effectiveness in standardizing result interpretation and facilitating large-scale opisthorchiasis screening campaigns.

The remainder of this paper is organized as follows. Section “Related works” explores AI techniques for diagnosing diseases using medical images and reviews the current state of using Information and Communication Technology (ICT) for mass screening campaigns. Section "Mass opisthorchiasis screening campaign" introduces the Mass Opisthorchiasis Screening Campaign and its implementation. Section “AI components” details the AI components of our platform, including the dataset preparation, two key subsystems (Image Quality Control and OV-RDT Grading), and their evaluation metrics and results. Section "AI models Implementation with cloud-based processing" describes the mobile application development and the usability testing outcomes. Section "Data pipeline processing" descritbes the data processing server architecture and implementation, including database infrastructure and data processing mechanisms. The data processing server is described in Section "Data pipeline processing". Section “Dashboard” elaborates on the dashboard development with its three main components: Overview Metrics, Geospatial Visualization, and OV-RDT Application Insight. Section "OV-RDT platform" presents the OV-RDT platform architecture and discusses five major screening campaigns conducted in northeastern Thailand, demonstrating the system’s effectiveness in real-world applications. Finally, we summarize our contributions and discuss future research directions in automated opisthorchiasis screening.

## Related works

The wealth of related work linked to the current work can be themed into two main topics.

1. AI techniques for diagnosing diseases.
2. Use of Information Communication Technology (ICT) for scaling mass screening.

### AI techniques for diagnosing diseases using medical images

Recent advances in AI-based disease diagnosis have been predominantly driven by deep learning, particularly convolutional neural networks (CNNs). Notable applications include Bellemo et al.^9^ study in Zambia, which utilized an ensemble of VGGNet and residual neural network architectures to detect diabetic retinopathy with clinically acceptable performance in population-based screening programs. Similarly, Cai et al.^10^ developed a computer-aided detection system using an 8-layer CNN to accurately identify early-stage esophageal squamous cell carcinoma through standard endoscopic imaging, significantly enhancing diagnostic accuracy from 81.70% to 91.10% and sensitivity from 74.20% to 89.20%. Several studies have demonstrated the effectiveness of transfer learning and pre-trained CNN models in medical image analysis. For instance, Chowdhury et al.^11^ achieved over 97.00% accuracy in detecting COVID-19 pneumonia from chest X-rays using pre-trained CNN models. Similarly, Mei et al.^12^ integrated CNN-based chest CT analysis with clinical symptoms and laboratory data to rapidly diagnose COVID-19 patients, achieving an area under the curve of 92.00%.

Beyond image-based diagnostics, AI has shown promise in disease detection through voice and speech analysis. Researchers have developed deep learning models that can detect various conditions through voice biomarkers. For example, Kaufman et al.^13^ demonstrated that machine learning models could identify type 2 diabetes with 70.00% accuracy based on voice samples, analyzing features like pitch intensity, and rhythm. Using smartphone-recorded voice segments, they found distinct vocal differences between diabetic and non-diabetic individuals, with features such as pitch variations in women and voice intensity in men serving as key indicators. Similarly, Almaghrabi et al.^14^ reviewed how bio-acoustic features could detect depression, with studies achieving accuracy rates of 80.00-87.00% by analyzing speech characteristics like pitch, loudness, and vocal perturbations.

In the broader field of computer vision, CNNs have revolutionized object detection and image quality assessment. For instance, He et al.^15^ introduced ResNet, which achieved groundbreaking performance on object detection tasks by introducing residual connections that enabled the training of much deeper networks. Building on this work, Lin et al.^16^ developed Feature Pyramid Networks (FPN) that significantly improved the detection of objects at different scales. In image quality assessment, pre-trained CNN models have shown remarkable capabilities in evaluating image degradation and artifacts. Madhusudana et al.^17^ demonstrated that deep learning models trained using contrastive learning could achieve competitive performance against state-of-the-art methods in evaluating image quality, even without requiring large labelled datasets. Their CONTRIQUE framework achieved 95.00% accuracy in detecting image quality status using self-supervised learning approaches. In contrast, our proposed OV-RDT platform takes a more targeted approach to image quality assessment, using the YOLOv5m model to detect and verify the precise positioning of the OV-Rapid test kit within a predefined camera template. This method achieved 98.00% accuracy in image quality estimation by focusing specifically on proper test kit placement and capture conditions, rather than evaluating general image quality characteristics. Our quality control algorithm ensures images meet the required standards for accurate AI grading by validating the position of the detected test kit bounding boxes against predetermined coordinates, with an acceptable margin of error of 3.00%.

In the context of COVID-19 rapid diagnostic tests, computer vision approaches have focused on efficient and accurate strip detection and interpretation. Mendels et al.^18^ developed a lightweight CNN-based system achieving 99.30% accuracy across 11 different RDT models, demonstrating that relatively simple convolutional architectures can effectively handle the structured nature of lateral flow tests. Similarly, Lee et al.^19^ introduced SMARTAI-LFA, a smartphone-based deep learning-assisted lateral flow assay (LFA) for the diagnosis of COVID-19, utilizing images from the LFA test kit to address the low accuracy of self-testing caused by human interpretation errors. They evaluated seven frameworks: DenseNet-121, DenseNet-161, ResNet-18, ResNet-34, ResNet-50, MobileNetV2 and SqueezeNet. RMSE values revealed that ResNet-18 and ResNet-50 were the most effective models, with ResNet-50 showing superior performance. However, due to longer computation times with deeper models, ResNet-18 was chosen. The CNN two-step model, which integrates YOLOv3 for object detection and ResNet-18 for classification, achieved 98% precision in various smartphones, outperforming both untrained users and human experts, especially in low-titer cases, where accuracy remained above 99%. As demonstrated by Arumugam et al.^20^, efficient CNN architectures with few layers can achieve robust performance while maintaining low computational overhead, making them ideal for resource-constrained settings like point-of-care testing. This aligns with the need for rapid, reliable, and accessible testing solutions that can run on standard mobile devices without requiring specialized hardware. This principle is exemplified in the work of Bermejo-Peláez et al.^21^ developed a smartphone-based AI platform to interpret and report SARS-CoV-2 rapid diagnostic tests (RDTs). Using MobileNetV2, the system analyzes standardized images of lateral flow immunoassays captured via a mobile app. Validated in nursing homes and a hospital emergency department, the model achieved high accuracy, with 100% sensitivity and 94.4% specificity for IgG detection, and correctly identified all antigen tests. By integrating AI-driven analysis with cloud-based monitoring, this solution enhances diagnostic reliability and epidemiological surveillance, particularly in low-resource environments.

For cholangiocarcinoma (CCA) screening, Intharah et al.^7^ developed BiTNet, a hybrid deep convolutional neural network model specifically designed for analyzing ultrasound images of the human biliary tract. The model serves dual purposes: as an auto-prescreening tool that reduces radiologists’ workload by accurately classifying images as normal or abnormal, and as an assisting tool that enhances diagnostic performance across healthcare professionals with varying experience levels. BiTNet’s architecture combines the strengths of both EfficientNet and DenseNet, incorporating residual connections and dense blocks to effectively capture complex biliary tract features. In validation studies, BiTNet demonstrated remarkable efficiency by reducing radiologist workload by 35.00% while maintaining an exceptionally low false negative rate of just 1 in 455 images. More importantly, when used as an assisting tool, it significantly improved the diagnostic accuracy and precision of healthcare professionals, with mean accuracy increasing from 50.00% to 74.00%. This improvement in diagnostic capability is particularly crucial in regions like northeastern Thailand, where CCA presents a significant public health challenge and early detection through ultrasound screening is a key strategy for improving patient outcomes.

In our work, we extend these advances in medical AI by proposing the OV-RDT platform, a comprehensive solution for mass opisthorchiasis screening that combines efficient CNN architectures with cloud computing infrastructure. Similar to BiTNet’s^7^ dual-purpose approach in CCA detection, our system serves both as an automated screening tool and a clinical decision support system. However, while BiTNet focuses on complex ultrasound image analysis, we adopt a more streamlined approach similar to COVID-19 RDT systems^18–20^, utilizing EfficientNet-B5 architecture for the relatively structured task of RDT interpretation. Our system achieves 95.00% accuracy in detecting OV infection status, comparable to the high accuracy rates seen in COVID-19 RDT systems, while maintaining computational efficiency for mobile deployment. Like Mendels et al.^18^, we emphasize quality control in image capture, incorporating a separate image quality assessment module to ensure reliable results. The platform’s cloud-based infrastructure enables real-time data collection and analysis across multiple screening locations, supporting over 100,000 participants across northeastern Thailand - a scale similar to successful screening programs like Bellemo et al.^9^ diabetic retinopathy detection system. This integration of efficient AI models with scalable infrastructure represents a significant advancement in parasitic infection screening capabilities, particularly crucial for regions where opisthorchiasis and subsequent CCA present major public health challenges.

### ICT solutions for mass screening campaigns

Beede et al.^22^ presented the deep learning system used in clinics to detection diabetic eye disease. In collaboration with Rajavithi Hospital, which operates under the Ministry of Public Health, a deep learning system is being implemented across numerous clinics across Thailand. This deployment is part of a significant prospective study involving 7,600 patients. The deep learning system was first used as part of the study in three clinics located in the province of Pathum Thani, namely Klong Luang, Nongsue, and Lamlukka. The three original sites conducted patient screenings from December 2018 to May 2019. After that, the sites’ eye screening season end, with a new one beginning in October 2019. Every clinic saw between 30 and 200 patients on screening days, and they provided screening for two to eight days a month. AIDR Deep learning systems are being employed at numerous clinics throughout Thailand in collaboration with Rajavithi Hospital, which is part of the Ministry of Public Health, Thailand. A deep learning algorithm-based method for DR assessment might lessen the need for nurses to grade in real-time and do away with the necessity for them to email fundus photos to an ophthalmologist for assessment. By clearing this bottleneck, nurses could refer patients more quickly, treat patients more quickly, and screen and review more cases. Additionally, patients will receive results immediately, allowing nurses to spend more time on diabetes education and management, ultimately improving patient outcomes. This project adopts AI models to improve efficiencies and reduce waiting times by instantaneously providing patients and healthcare workers with test results. Our system also utilizes the AI to shorten the interval between conducting tests and receiving results in relevant databases and for stakeholders.

Lauritzen et al.^23^ introduced the screening process focused on detecting breast cancer among women. The screening protocol involves the use of artificial intelligence (AI) technology to analyze mammograms and identify potential signs of breast cancer. This AI-based approach aims to assist radiologists in interpreting mammograms more efficiently and accurately. The study involved 114,421 asymptomatic women aged 50–69 years old in the Capital Region of Denmark participating in the campaign. This technology has the potential to decrease the workload of radiologists by 62.60%, significantly. (Mammograms from 71,585 of 114,421 screenings were read only by the AI system), and 25.10% (AI-based screening, 529 out of 2,107 false-positive screenings were avoided). Implementing an AI-based reading protocol in clinics could improve screening outcomes by decreasing the number of mammograms requiring radiologist interpretation, enhancing the accuracy of AI-driven diagnoses, speeding up the processing of mammograms, and reducing both false positives and negatives. The study also evaluates the broader impact of the screening campaign on various factors related to breast cancer detection and healthcare delivery. This includes insights into early detection rates, improvements in patient outcomes, cost-effectiveness, radiologist productivity, and potential enhancements in healthcare resource allocation based on screening results.

Pantanowitz et al.^24^ proposed a screening process for detecting prostate cancer in patients by developing a high-calibre AI-based algorithm for evaluating digitalized prostate core needle biopsy (CNB) slides and successfully integrating it into standard clinical procedures. The proposed system demonstrated remarkable accuracy in detecting perineural invasion in CNBs, diagnosing and quantifying prostate cancer, and effectively distinguishing between low-grade and high-grade tumors. This study reports the successful development, external clinical validation, and deployment in clinical use by Maccabi Healthcare Services, a large healthcare provider in Israel with approximately 120,000 surgical pathology cases annually. Around 40.00% of these CNBs are diagnosed with cancer. Galen Prostate (Ibex Medical Analytics), a prostate algorithm-based tool, has been utilized by the Maccabi Pathology Institute since March 2018.

Khuntikeo et al.^25^ proposed a screening method aimed at the early diagnosis of cholangiocarcinoma (CCA). To facilitate this, Khon Kaen University has established the “Cholangiocarcinoma Screening and Care Program (CASCAP)” in collaboration with the National Health Security Office (NHSO) and the Ministry of Public Health in Thailand. These organizations have implemented a policy aimed at enhancing the systematic diagnosis and treatment of individuals with CCA across the northeastern region of Thailand. This study focuses on individuals who have tested positive for liver fluke and are at risk of developing CCA, as well as those who have already been diagnosed with the disease. The objective is to ensure these groups receive appropriate treatment from public health experts. This study focuses on individuals who have tested positive for liver fluke and are at risk of developing CCA, as well as those who have already been diagnosed with CCA. The objective is to ensure that all these groups receive appropriate treatment from public health experts. Currently, ultrasonography is the standard method for screening biliary system disorders. Ultrasonographic evaluation is based on disease pathology and the density of OV eggs in stool samples^5,6^. The strategy aims to provide long-term screening and follow-up for 150,000 individuals within the at-risk population, which numbers approximately 20 million in Thailand. As of September 5, 2023, the CASCAP database has recorded 3,604,546 examined subjects. While CASCAP’s digital infrastructure accumulates and stores valuable data, the detection process relies heavily on manual testing, which is resource-intensive and presents challenges to scalability. In response to these challenges, the OVRDT system draws inspiration from CASCAP to digitize and manage data through a cloud-based infrastructure, significantly enhancing scalability and ease of maintenance. Additionally, our system incorporates cutting-edge AI technologies within the detection layer, addressing the pain points of CASCAP that currently limit its ability to scale effectively.

## Mass opisthorchiasis screening campaign

In addition to ultrasound screening, detecting individuals infected with *Opisthorchis viverrini* shows significant potential to rapidly identify at-risk populations in areas where the infection is prevalent. Worasith et al.^8^ developed a urinary antigen-based rapid diagnostic test (RDT) to simplify the diagnosis of opisthorchiasis, making it suitable for point-of-care testing (POCT) in field applications for disease surveillance and control. The overall sample population consisted of 101,821 individuals, all residents from subdistricts across 21 provinces in northeastern and northern Thailand. An AI component was developed to assist in interpreting the test results. The OV-RDT Platform includes Android/iOS mobile applications, an AI server, a data processing server, and an interactive dashboard. The OV-RDT mobile application is specifically designed for healthcare professionals to capture images of the OV-RDT and collect relevant data.

**Fig. 1:**
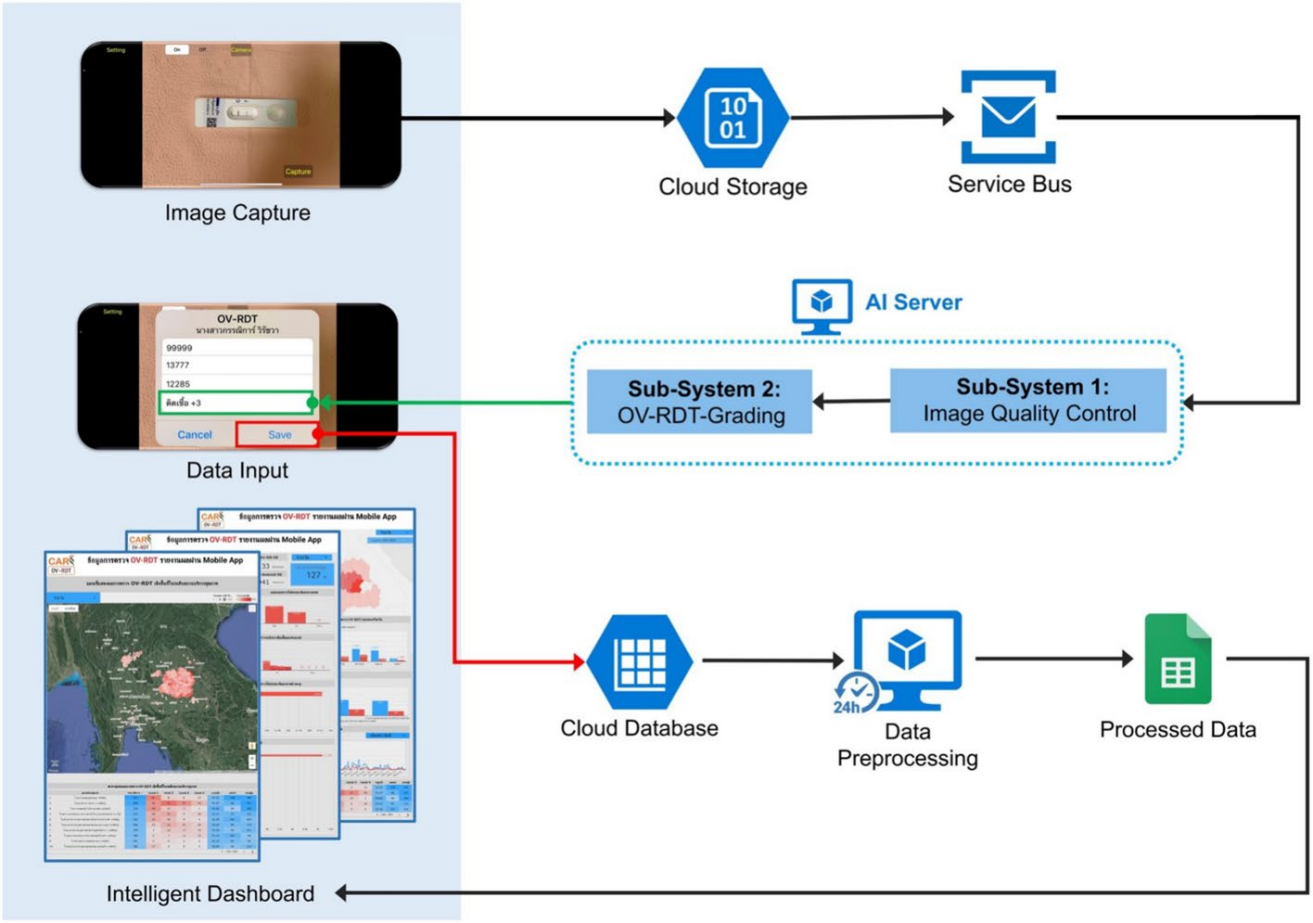
System architecture of the OV-RDT platform composes of three main components: (1) mobile application interface for image capture and data input across Android and iOS devices, (2) AI server with image quality control and OV-RDT grading subsystems for diagnostic analysis, and (3) data processing infrastructure including cloud database, data preprocessing, and intelligent dashboard for results visualization. All maps in the figure are originally generated by the authors through https://cloud.google.com/looker-studio. The satellite image is from Google Imagery.

In the screening process, the AI server retrieves tasks from the service bus queue to evaluate the image quality control of the input images. Once an image meets the established criteria, it is forwarded to the OV-RDT grading. The data processing server generates reports that links the screening data with patient information before sending the data to the cloud database. The interactive dashboard processes the data for convenient access and prompt usage, displaying summarized information from OV-RDT grading reports and application usage statistics. The OV-RDT mobile application can significantly enhance the interpretation of results and the reporting of opisthorchiasis in mass screening efforts. This approach allows for real-time data processing and eliminates the need for paper storage. The ease of sample collection and handling makes the urinary OV-RDT a promising point-of-care testing (POCT) method to facilitate large-scale screening and control efforts aimed at eliminating opisthorchiasis. This will ultimately reduce the number of cholangiocarcinoma (CCA) cases in Southeast Asia and the Lower Mekong Basin countries. Overview of architecture of the OV-RDT platform is illustrated in Fig. 1.

### Ethics statement

This study was conducted according to the guidelines of the Declaration of Helsinki and approved by the Human Ethics Committee of Khon Kaen University, Khon Kaen, Thailand, based on the ethics of human specimen experimentation of the National Research Council of Thailand (HE664027; approved date August 30, 2023). Ethics approval HE664027 was obtained as an expedited review from the primary research project number HE631572. informed consent was obtained from all subjects and/or their legal guardian(s). While we utilized only image data for this study, the informed consent process for all volunteers was thoroughly conducted through the main research project HE631572. This primary project had already established comprehensive ethical protocols including proper informed consent procedures, data privacy protection measures, and participant anonymity as required by the Human Ethics Committee of Khon Kaen University and in accordance with the Declaration of Helsinki. Confidentiality and data privacy were rigorously upheld throughout the research process to ensure the participant’s well-being and privacy. Furthermore, the survey was conducted anonymously and all data was analyzed anonymously.

## AI components

This section delves into the artificial intelligence (AI) components integral to the OV-RDT platform. We describe the dataset used for training and evaluation, while describing two main AI subsystems—1) image quality control and 2) OV-RDT grading. We also explain the evaluation metrics and the experimental design employed to validate the performance of these AI subsystems. Finally, the results, discussion, and effectiveness of the AI implications in improving diagnostic accuracy are presented.

### Dataset

In this experiment, we utilized a dataset comprising 8,624 OV Rapid Test Kit images, all meticulously collected and annotated by experts to ensure high data quality. The dataset was divided based on a train-test split criterion: 6,900 images were allocated for training the model, while the remaining 1,724 images were reserved for testing. This approach ensured that the model’s performance could be evaluated on a separate, unseen set of images, providing a reliable measure of its generalization capability and accuracy in real-world scenarios. The dataset was collected during the Cholangiocarcinoma Research Institute (CARI) outreach between 18 February and 1 June, 2024. To ensure experimental integrity and prevent data leakage, this train-test split was performed once at the beginning of the study and maintained consistently across all experiments. The test set of 1,724 images was never exposed to any model during training or hyperparameter optimization phases. We named it the OV-RDT classification dataset which only contains good quality strip images. The demographic distribution of the dataset is summarized in Table 1.

Additionally, we collected 100 images of OV Rapid Test Kits labelled as failed capture images during the application’s image capture process. These images were specifically used to evaluate the image quality control subsystem. We also randomly selected 100 successful capture images from the existing OV-RDT Classification dataset. This combination created a balanced dataset, allowing us to thoroughly assess the model’s performance in estimating image quality across various capture conditions. In addition to the main classification dataset, we collected a separate dataset for training the image quality control system. This comprised 574 OV Rapid Test Kit images captured through dedicated data collection sessions designed to include various capture conditions, positioning errors, and lighting scenarios. These images were systematically annotated with bounding boxes for the YOLOv5m object detection model training, ensuring a well-balanced dataset for developing robust quality control capabilities.

**Table 1:** Demographic characteristics of the participants found in the OV-RDT classification dataset.

Sex	Mean Age	Number of Images	Level 0 (Not Detected)	Detected			
				Level 1	Level 2	Level 3	Level 4
Female	53.22	5,158	921	1,089	1,091	1,006	1,051
Male	55.28	3,207	628	612	731	627	609
Unspecified	56.58	259	124	56	36	27	16
**Total**		**8,624**	**1,673**	**1,757**	**1,858**	**1,660**	**1,676**

### AI sub-system 1: image quality control

This AI-based module’s advantage lies in its ability to locate the OV Rapid Test Kit within images collected from the OV-RDT mobile application. The YOLOv5m model was utilized as the baseline architecture for detecting the OV Rapid Test Kit in images. As a medium-sized model within the YOLOv5 family, YOLOv5m is well-regarded for real-time object detection tasks with a good balance of accuracy and speed. For model training, we utilized a dedicated dataset of 574 annotated OV Rapid Test Kit images collected through controlled data collection sessions using our OV-RDT mobile application (both Android and iOS versions) during the early development phase (January-February 2024). This dataset was specifically designed to be well-balanced for machine learning model development, separate from the main OV-RDT classification dataset, ensuring representation of various capture conditions and positioning scenarios. From these 574 images, 479 were used for training, while the remaining 95 images were allocated for validation. All experiments were conducted on a workstation equipped with two NVIDIA GeForce RTX 2080 Ti GPUs. It is important to note that the dataset used for training the YOLOv5m image quality control model (574 images) was completely separate from the OV-RDT grading dataset (8,624 images), ensuring no data leakage between these two subsystems.

The system integrates a verification step known as the quality module, which is described in Algorithm 1. This module verifies the correctness of the test kit’s positioning within the camera template by checking the location of the detected bounding box and comparing it with predefined spatial constraints. If the detected box deviates from the expected location by more than a specified margin, the system prompts the user to retake the image.

The YOLOv5m model was fine-tuned with a specific set of hyperparameters designed to optimize detection accuracy for the OV-RDT domain. The training configuration is detailed as follows:

- **Learning Rate:** We utilized an initial learning rate of 0.001 with OneCycleLR scheduling, decreasing to 0.0001 ($lr0 \times lrf = 0.001 \times 0.1$) over 400 epochs. A warmup period of 3 epochs was applied with a warmup bias learning rate of 0.1.
- **Batch Normalization:** Applied across all layers with a momentum coefficient of 0.937.
- **Optimizer:** The model was optimized using Stochastic Gradient Descent (SGD) with momentum = 0.937 and weight decay = 0.0005, including an initial warmup momentum of 0.8.
- **Batch Size:** Training was performed with a batch size of 16 using input images resized to 640$\times$640 pixels.
- **Augmentation:** Various data augmentation techniques were used, including mosaic augmentation (probability = 1.0), random scale transformations (±0.5), translation adjustments (±0.1), and HSV color space modifications (hue ±0.015, saturation ±0.7, value ±0.4).
- **Loss Function:** A composite loss function was employed, comprising box loss (gain = 0.05), classification loss (gain = 0.5), and objectness loss (gain = 1.0), with an IoU training threshold of 0.20 and anchor-multiple threshold of 4.0.

This training setup enabled robust and consistent detection of OV test kits across a wide variety of image capture conditions, laying the foundation for reliable downstream grading and classification tasks in the OV-RDT platform.

**Algorithm 1:**
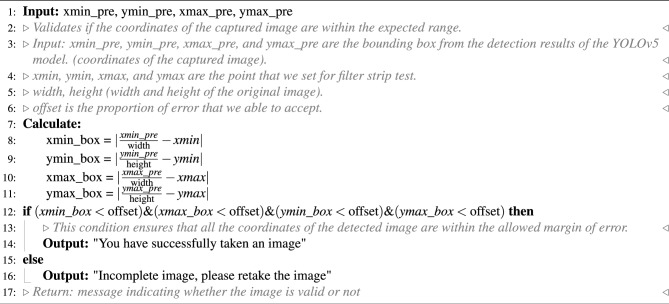
OV-RDT strip image quality control algorithm.

Algorithm 1 requires four parameters: $xmin\_pre$, $ymin\_pre$, $xmax\_pre$ and $ymax\_pre$ (bounding box). These four parameters represent the bounding box of the detected OV-Rapid test kit. The margin of the bounding box is compared to that of the predefined bounding box characterised by $xmin = 0.271307$, $ymin = 0.389985$, $xmax = 0.780217$ and $ymax = 0.589000$. Then, we compute the margin error between the detected bounding box and the predefined one. Images are considered successfully capturedif the margin error is less than or equal to the offset value ($offset = 0.03$); otherwise, it is classified as a failed capture. The combination of standardized image capture through the camera template and YOLOv5m-based detection creates a robust quality control system. Images with poor quality characteristics such as blur or inadequate lighting naturally fail the detection process, as the YOLO model’s confidence drops below the acceptance threshold in these cases.

**Fig. 2:**
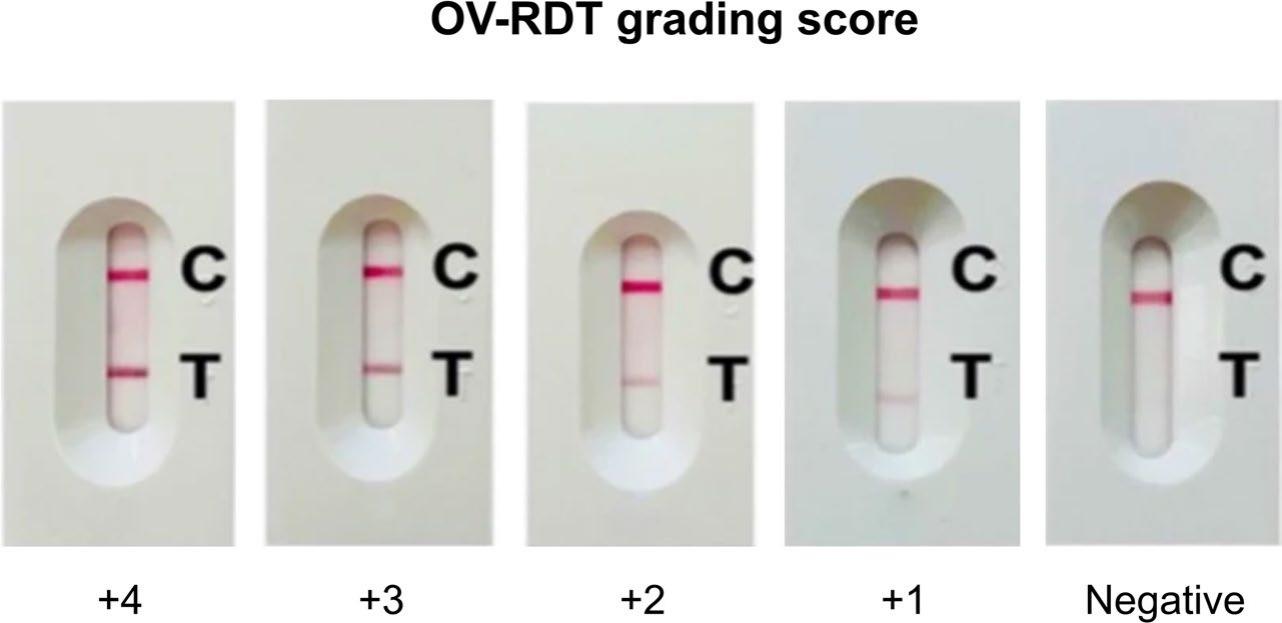
The standard OV-RDT color chart for the score based on the band intensity from $+1$ to $+4$ was constructed using *Opisthorchis viverrini* crude antigen diluted in clean urine. The color intensity at T was expressed as $+4$ for the highest intensity and $+1$ for the lowest intensity, implying lover of infection.

### AI sub-system 2: OV-RDT grading

We employed artificial intelligence to predict OV-RDT grading scores ranging from 0 to +4, as illustrated in Fig. 2. The grading scores correspond to two diagnostic statuses: Negative (score 0) and Positive (scores +1, +2, +3, or +4). Our experimental dataset comprised 8,624 expert-annotated images of OV Rapid Test Kits, divided into 6,900 training images and 1,724 testing images. All images underwent quality control using YOLOv5m before model training. We evaluated multiple machine learning approaches for both multi-class grading (five levels: 0, +1, +2, +3, +4) and binary classification (Negative vs. Positive). EfficientNet-B5 was selected as the primary deep learning architecture due to its optimal balance between model size and accuracy, making it particularly suitable for mobile deployment. We benchmarked this against other proven architectures, including ResNet50^15^ and MobileNetV2^26^ for both classification and regression tasks, providing insights into performance trade-offs across different neural network architectures for OV-RDT interpretation.

**Fig. 3:**
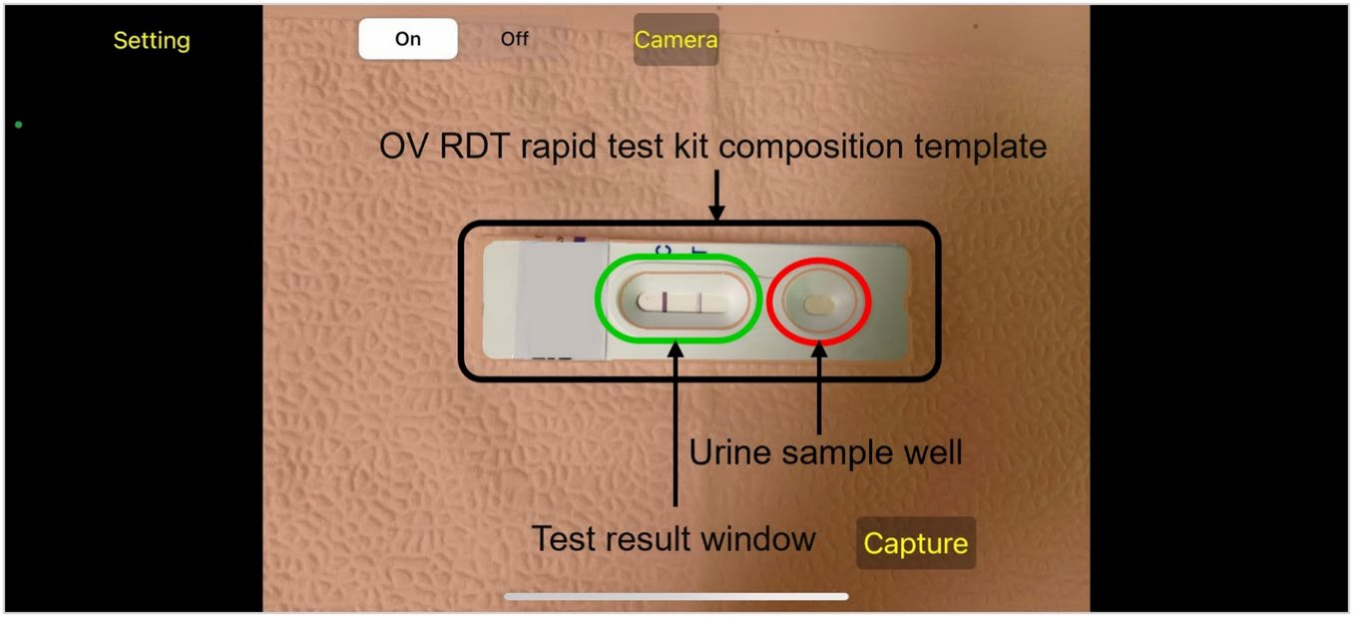
An example image when placing the OV-Rapid test kit within a camera template to ensure image quality on both positioning and distance between the strip and the camera.

Additionally, we implemented traditional machine learning models—Random Forest^27^ and Support Vector Machine (SVM)^28^ for multi-class classification. These models utilised feature vectors derived from red (R), green (G), and blue (B) colour values extracted from the test result area, following the methodology of DentShadeAI^29^. We specifically focused on the green channel, which provided optimal contrast for T-band detection as shown in Fig. 3. Both traditional models were trained using the Scikit-Learn package^30^ with five-fold cross-validation for hyperparameter optimisation. The five-fold cross-validation for Random Forest and SVM models was conducted exclusively within the training set of 6,900 images, with the test set of 1,724 images held out for final evaluation only.

The EfficientNet-B5 training was conducted using TensorFlow with pre-trained ImageNet weights and executed on a workstation equipped with dual NVIDIA GeForce RTX 2080 Ti GPUs. A two-phase transfer learning strategy was employed: in the first phase, all convolutional layers were frozen and only the classification head was trained for 200 epochs. In the second phase, layers from Block 5 onward were unfrozen and fine-tuned for an additional 200 epochs, allowing the model to adapt to OV-RDT-specific features. A batch size of 16 was used throughout, with RMSProp selected as the optimizer, an initial learning rate of $2 \times 10^{-5}$, and categorical cross-entropy as the loss function (mean squared error was used in regression experiments). To enhance model generalization and mitigate overfitting, a variety of data augmentation techniques were applied, including random rotation ($\pm 10^\circ$), zoom ($\pm 10\%$), brightness and contrast shifts ($\pm 20\%$), and random cropping and resizing. Horizontal flipping was excluded due to the direction-sensitive layout of the test kits. Regularization techniques included L2 weight decay ($1\times 10^{-5}$), dropout (rate = 0.3), early stopping (patience = 15 epochs), and adaptive learning rate reduction via ReduceLROnPlateau (factor = 0.5 after 5 stagnant epochs). On average, training required approximately 12 hours per model. The final model size was approximately 119 MB, and inference time was around 60 ms per image, supporting real-time performance on mobile devices. This training pipeline enabled EfficientNet-B5 to deliver high performance across both fine-grained multi-class grading and binary OV infection classification in real-world screening scenarios. While color normalization and stain standardization techniques have shown promise in medical image analysis, we opted to rely on the robustness of pre-trained models and our standardized image capture protocol for this initial implementation. Future work will investigate preprocessing methods specifically tailored to RDT strip analysis to potentially enhance grading accuracy, particularly for cases with subtle T-band intensity differences.

For model interpretability, we implemented GradCAM using the TensorFlow GradCAM library, extracting activation maps from the final convolutional layer ($top\_conv$) of our fine-tuned EfficientNet-B5 model. The gradients were computed with respect to the predicted class score, and the resulting heatmaps were resized to match the input image dimensions (224×224) using bilinear interpolation.

### Evaluation metrics and experimental design

We used a confusion matrix to evaluate the performance of the OV infection classification model. The evaluation was conducted on a test set comprising 1, 724 images, all of which had passed the image quality control step. The confusion matrix is a standard tool for analyzing classification models, providing a detailed breakdown of prediction outcomes by comparing predicted labels with ground-truth annotations. It reports results in terms of four key categories: true positives (TP), where positive cases are correctly identified; true negatives (TN), where negative cases are correctly classified; false positives (FP), where negative cases are incorrectly predicted as positive; and false negatives (FN), where positive cases are missed by the model. This categorization offers insight into not only overall model performance but also the types of errors that occur.

Based on the confusion matrix, we computed four performance metrics: accuracy, precision, recall, and F1-score. Accuracy represents the proportion of correct predictions among all predictions made. Precision measures the proportion of true positives among all predicted positives, reflecting the model’s ability to avoid false alarms. Recall (or sensitivity) indicates the model’s ability to detect actual positive cases, which is particularly critical in medical screening applications where missing a positive case can have serious consequences. The F1-score combines precision and recall into a single metric, providing a balanced measure of the model’s performance, especially in cases where the class distribution is imbalanced. Together, these metrics offer a comprehensive evaluation of the model’s reliability in real-world OV-RDT classification scenarios.

To evaluate the performance of the image quality control module, we used a dataset of 200 OV-RDT images, comprising 100 successful captures and 100 failed ones, as labeled by domain experts. The model’s performance was measured using accuracy, precision, and recall. To further analyze its behavior, we categorized the results into four outcome types. True positive cases refer to instances where the test kit was properly placed within the camera frame and was correctly detected. These images typically featured good lighting, centered alignment, and correct orientation. True negative cases occurred when the test kit was improperly positioned, for example skewed, off-center, or blurred, and the model correctly rejected them. False positives describe cases where the model accepted an image that was not properly positioned, often due to slight misalignment or uneven lighting. False negatives occurred when the test kit was placed correctly but the model failed to detect it, usually because of glare, background interference, or poor contrast. This classification provides insight into the model’s strengths and the types of errors that can arise under real-world image capture conditions.

To establish a robust statistical framework for classification evaluation, we systematically partitioned the test dataset into 30 non-overlapping subsets (n$\approx$60 samples per subset) using randomized sampling without replacement. Model performance was quantified across four complementary metrics: accuracy, precision, recall, and F1-score. For each metric, we calculated both the sample mean and standard deviation across all subsets. In accordance with the central limit theorem, we verified that the sampling distributions of performance metrics approximated normal distributions. Homogeneity of variance across the eight methods was confirmed via Levene’s test prior to conducting inferential analyses. We then employed one-way ANOVA to test the null hypothesis of equal mean performance across methods. Where the ANOVA null hypothesis was rejected (p < 0.05), we performed Tukey’s Honest Significant Difference (HSD) post-hoc analysis to identify statistically significant pairwise differences between methods while controlling the familywise error rate. Additionally, we utilized Cohen’s Kappa coefficient to quantify the reliability of our model’s predictions across the spectrum of infection intensity levels and grading status. This chance-corrected measure of agreement is particularly valuable when evaluating a graded classification system such as ours, where test line intensity directly corresponds to OV antigen concentration. Since the visual markers range from weak (1+) for low antigen levels to intense (4+) for high parasite loads, Cohen’s Kappa provides statistical validation of the model’s ability to discern these subtle gradations consistently. This metric offers more clinical relevance than standard accuracy measures alone, as it specifically evaluates the system’s reliability in distinguishing between different degrees of infection severity, which is critical for appropriate treatment planning and epidemiological monitoring.

To assess the clinical implications of model performance, we analyzed the confusion matrix to identify patterns in misclassification, particularly focusing on clinically significant errors (e.g., false negatives in binary classification or misclassification by more than one grade level in multi-class grading). The moderate Kappa score (0.55) for multi-class grading reflects the subjective nature of visual intensity assessment, a known challenge in immunochromatographic test interpretation that our platform addresses through human oversight and adaptive threshold management.

### Results and discussion

**Fig. 4:**
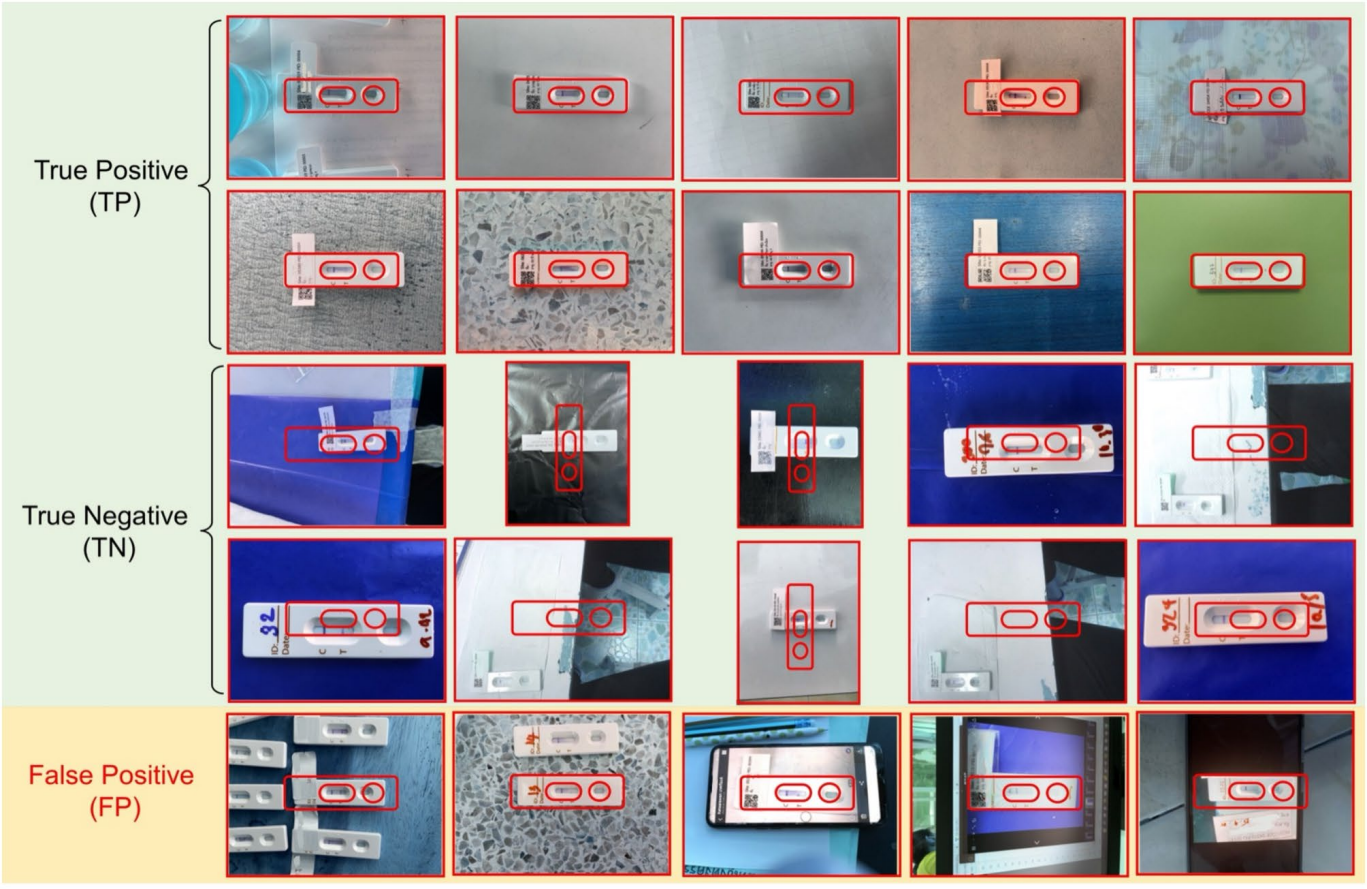
Examples of results from the OV-RDT strip image quality control algorithm: True Positive indicates the examples that the algorithm accurately classify as good quality images, True Negative indicates the examples that the algorithm accurately classify as poor quality images, and False Positive indicates the examples that the algorithm mis-classify as good quality images.

The EfficientNet-B5 model for image quality control exhibited outstanding performance in assessing image quality. The model achieved accuracy, precision, and recall scores of 98.00%, 95.00%, and 100.00%, respectively, indicating high reliability. An accuracy of 98.00% reveals that the model accurately classified the vast majority of images, while a precision of 95.00% indicates that nearly all images predicted to be of high quality were indeed high quality. The perfect recall score of 100.00% also underscores that the model successfully identified all high-quality images, capturing all relevant cases. The high performance of our quality control approach (98% accuracy) validates that the YOLOv5m model, when combined with our fixed capture template and spatial verification algorithm, provides sufficient quality assurance for downstream classification tasks. This approach effectively filters out images with quality issues including blur, poor lighting, and improper positioning without requiring additional pre-processing steps. The effectiveness of the model is further illustrated in Fig. 4, where examples of true positive, true negative, and false positive images are presented. These examples highlight how the model distinguishes between correctly identified high-quality images (true positives), correctly identified low-quality images (true negatives), and cases where the model mistakenly classified low-quality images as high-quality (false positives). This high level of performance makes the EfficientNet-B5 model an excellent choice for practical applications in automated image quality assessment.

**Fig. 5:**
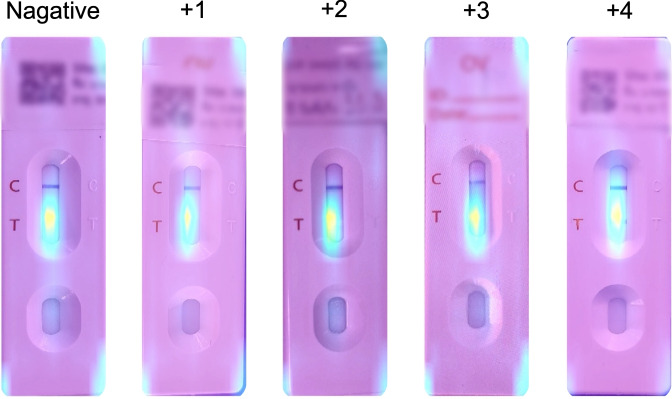
GradCAM visualization showing model attention patterns for OV-RDT grading. Representative heatmaps for each infection grade (0 to +4) demonstrate that the model correctly focuses on the T-band region, with activation intensity correlating with infection severity. Warmer colors (red/yellow) indicate regions of higher importance for the model’s decision.

**Table 2:** Comparison of different machine learning models on the performance in estimation OV infectious level grading. The best performance and the next best performance are indicated in **bold** and *italic* respectively. $\circ$ represents a classification model and $\triangle$ represents a regression model. The proposed models that outperform the most performant baseline, ResNet50 $\triangle$ ($p <0.05$) are marked with $*$.

Task	Models	Performance (± s.d)			
		Accuracy	Recall	Precision	F1-score
OV-RDT grading (0, +1, +2, +3, +4)	SVM $\circ$	0.48 ± 0.07	0.48 ± 0.07	0.50 ± 0.06	0.48 ± 0.07
	RF $\circ$	0.44 ± 0.06	0.44 ± 0.06	0.46 ± 0.07	0.44 ± 0.06
	MobileNetV2 $\circ$	0.48 ± 0.05	0.48 ± 0.05	0.49 ± 0.06	0.46 ± 0.05
	MobileNetV2 $\triangle$	0.55 ± 0.06	0.55 ± 0.06	0.59 ± 0.06	0.55 ± 0.06
	ResNet50 $\circ$	0.58 ± 0.07	0.58 ± 0.07	0.57 ± 0.09	0.57 ± 0.07
	ResNet50 $\triangle$	0.58 ± 0.07	0.58 ± 0.07	0.61 ± 0.08	0.58 ± 0.07
	EffNet-B5 $\circ$ (Ours)	*0.63* ± *0.06*	*0.63* ± *0.06*	*0.64* ± *0.06*	*0.62* ± *0.06*
	EffNet-B5 $\triangle$ (Ours)	**0.66**$\pm {\textbf {0.06}}^*$	**0.66**$\pm {\textbf {0.06}}^*$	**0.68**$\pm {\textbf {0.06}}^{*}$	**0.66**$\pm {\textbf {0.06}}^{*}$
OV-RDT Status (Negative (0), Positive (1–4))	SVM $\circ$	0.84 ± 0.04	0.84 ± 0.04	0.87 ± 0.04	0.85 ± 0.04
	RF $\circ$	0.78 ± 0.04	0.78 ± 0.04	0.81 ± 0.04	0.79 ± 0.04
	MobileNetV2 $\circ$	0.87 ± 0.04	0.87 ± 0.04	0.91 ± 0.03	0.88 ± 0.04
	MobileNetV2 $\triangle$	0.91 ± 0.04	0.91 ± 0.04	0.92 ± 0.03	0.91 ± 0.03
	Resnet50 $\circ$	0.90 ± 0.04	0.90 ± 0.04	0.93 ± 0.02	0.90 ± 0.03
	ResNet50 $\triangle$	0.92 ± 0.03	0.92 ± 0.03	0.93 ± 0.03	0.92 ± 0.03
	EffNet-B5 $\circ$ (Ours)	*0.95*$\pm 0.03 ^{*}$	*0.95*$\pm 0.03 ^{*}$	*0.95*$\pm 0.03 ^{*}$	*0.95*$\pm 0.03 ^{*}$
	EffNet-B5 $\triangle$ (Ours)	**0.95**$\pm {\textbf {0.03}}^*$	**0.95**$\pm {\textbf {0.03}}^{*}$	**0.96**$\pm {\textbf {0.03}}^{*}$	**0.95**$\pm {\textbf {0.03}}^{*}$

Table 2 presents the experimental results for classification tasks. The sample mean plus/minus standard deviation across eight methods was computed for each performance metric. Since the null hypothesis of the ANOVA test was rejected, the Multiple Comparisons of Means - Tukey HSD analysis was conducted to differentiate the performance across eight state-of-the-art methods statistically. EffNet-B5-based model for regression demonstrated clear statistical superiority over most competing models (p<0.05) based on Tukey HSD analysis. It achieved the highest performance across all metrics (0.66 ± 0.06), significantly outperforming ResNet50 variants, MobileNetV2 variants, SVM, and RF. The significant performance gap between EffNet-B5 R and the other based line models, including ResNet50, MobileNetV2, SVM, and RF, highlight its effectiveness for this complex multi-class problem. The performance pattern changed notably for the simpler OV-RDT status task (binary classification). The proposed EffNet-B5-based models achieved identical top performance (0.95 ± 0.03) with no statistically significant difference (both marked with superscript *). They significantly outperformed all other models, with ResNet50 and MobileNetV2 variants and still delivered strong results. Furthermore, the Kappa scores reveal important insights about model reliability given in Table S1 in the supplementary material. For the more complex grading task (5-class classification), EffNet-B5 R achieves the highest Kappa score (0.55), indicating moderate agreement beyond chance. Despite high accuracy metrics, this moderate score suggests the multi-class task’s challenging nature. For the binary status classification, substantially higher Kappa scores are observed across all models, with EffNet-B5 R leading at 0.83, indicating strong agreement. The consistent pattern of lower Kappa values for traditional machine learning methods (SVM: 0.37/0.49, RF: 0.29/0.34) compared to deep learning approaches confirms that neural network architectures provide more reliable classification performance in both contexts, with EffNet models demonstrating the most consistent reliability across both classification tasks. The details of the ANOVA test and Tukey’s Honest Significant Difference (HSD) post-hoc analysis results can be found in Tables S2-S9 in the supplementary material.

### Model interpretability analysis

To enhance the transparency and trustworthiness of our AI system, we implemented Gradient-weighted Class Activation Mapping (GradCAM) to visualize the regions of OV-RDT images that most influence the model’s grading decisions. GradCAM generates visual explanations by examining the gradients flowing into the final convolutional layer of our EfficientNet-B5 model, highlighting the discriminative regions used for classification.

We applied GradCAM analysis to a representative subset of 100 test images across all five grading levels. The resulting heatmaps were overlaid on the original OV-RDT images to identify which spatial regions contributed most significantly to the model’s predictions. As shown in Fig. 5, the GradCAM visualizations demonstrate that our model correctly focuses on the T-band (test line) region when determining infection grades. The intensity of activation in the T-band area progressively increases from grade 0 (negative) through grade +4, corresponding to the visual intensity of the test line itself.

This analysis provides important validation that our model has learned to identify clinically relevant features rather than relying on spurious correlations or image artifacts. The concentrated attention on the T-band region aligns with expert diagnostic practices, where the color intensity of this specific area directly indicates the level of OV antigen present in the urine sample.

## AI models implementation with cloud-based processing

Implementing AI diagnostics through a mobile application interface with cloud-based processing offers significant advantages for OV-RDT interpretation in clinical settings. This architecture leverages widely available smartphone cameras for image acquisition while utilizing powerful cloud servers for computationally intensive model execution. Healthcare workers can capture standardized images of rapid diagnostic tests using the mobile application’s guided interface, transmit these securely to cloud infrastructure where the EffNet-B5 R model resides, and receive expert-level interpretations within seconds. This approach eliminates the subjective variability inherent in visual RDT interpretation while maintaining accessibility at the point of care. The mobile-to-cloud implementation creates a sustainable infrastructure for diagnostic AI that balances accessibility with computational power, making sophisticated OV-RDT grading available wherever basic connectivity exists while maintaining the high performance demonstrated by the EffNet-B5 R model in statistical evaluations.

### Mobile application development process

The development of the OV-RDT mobile application followed a comprehensive, user-centered approach tailored specifically for diagnostic use in healthcare settings. The process began with thorough requirements gathering from domain experts, including parasitologists, medical technologists, and researchers from the Cholangiocarcinoma Research Institute (CARI) at Khon Kaen University in Thailand. The multidisciplinary collaboration ensured the application would address real clinical needs and align with existing liver fluke infection screening protocols. The development team structured the application around four core functional requirements:

- Image Capture Aiding: Implementation of a camera template with an AI-based quality validation module to ensure consistent, high-quality images of OV-RDT test kits, critical for accurate analysis.
- Rapid Data Input and Patient Information Retrieval: Integration of QR-code functionality to efficiently retrieve patient information from hospital databases, streamlining the workflow and reducing manual data entry errors.
- AI-based Image Grading: Development of an advanced classification system capable of categorizing OV-RDT results into five severity levels, leveraging the cloud-based EffNet-B5 R model identified as optimal through statistical testing. The AI suggestions are presented as default values that healthcare workers can review and modify, ensuring human oversight for borderline cases where automated grading confidence is lower.
- Epidemiological Analytical Features: Creating a real-time intelligent dashboard displaying both user inputs and AI-generated diagnostic results to support broader public health monitoring and intervention planning.

**Fig. 6:**
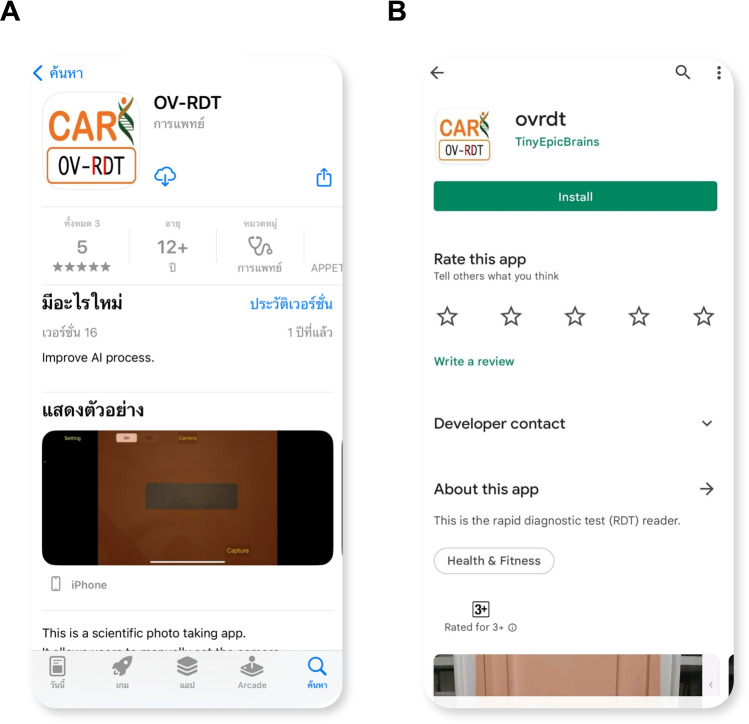
The OV-RDT mobile application. (**A**) Application for the Android operating system. (**B**) Application for the iOS operating system.

The application was developed cross-platform for Android and iOS to maximize accessibility and deployed through official channels (Google Play Store and App Store, as shown in Figure 6). Before release, the application underwent rigorous testing on current operating systems (Android 14.0 and iOS 17.4.1) to ensure compatibility and performance. This methodical development process resulted in a comprehensive diagnostic tool that serves individual patient needs and contributes to regional epidemiological surveillance for liver fluke infections.

For usability assessment, the proposed mobile application employed a rigorous cross-sectional methodology conducted during World Cholangiocarcinoma Day in February 2024 at Khon Kaen University, Thailand. The evaluation targeted 50 volunteer expert workers actively involved in liver fluke disease screening, using a structured online questionnaire that measured both satisfaction with the OV-Rapid test kit and the mobile application. The mobile application assessment specifically examined four key dimensions: user satisfaction, expectations for future use, obstacles encountered during application usage, and additional user suggestions. Data was collected using a standardized 5-point Likert scale^31^ ranging from 1 (Dissatisfied) to 5 (Extremely Satisfied), with precisely defined interpretation intervals (e.g., 4.21-5.00 indicating ”Extremely Satisfied”), ensuring consistent evaluation metrics across participants and facilitating quantitative analysis of the user experience among healthcare professionals directly involved in liver fluke screening.

### Data pipeline processing

**Fig. 7:**
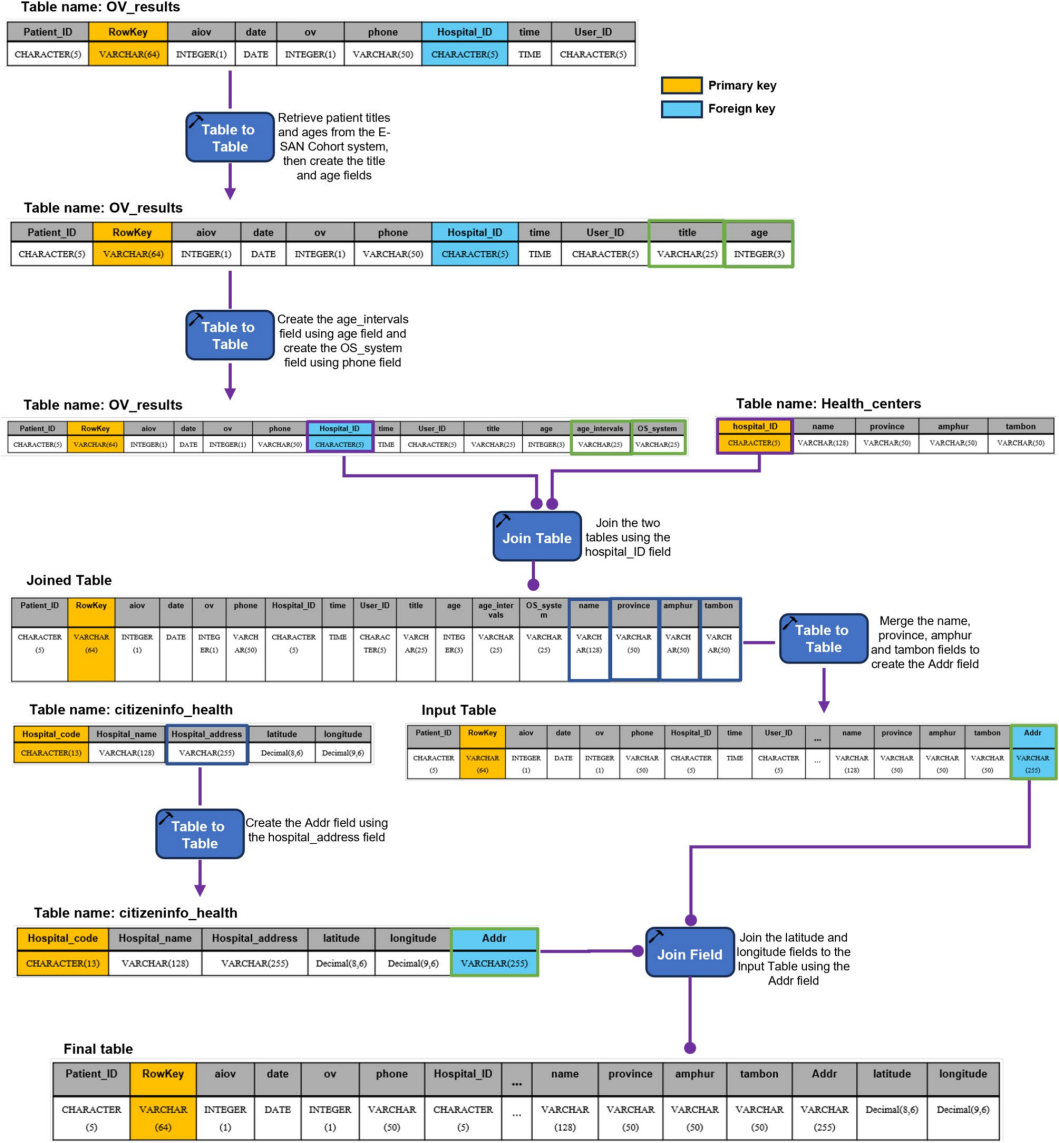
The data integration process combines data from three import tables into the final table. The final table is used as a data warehouse that feeds data to the interactive dashboard visualization in the next step.

AI-based diagnostics leverages Azure Cloud infrastructure to create a comprehensive end-to-end diagnostic pipeline optimized for liver fluke screening. As detailed in Fig. 1, the workflow begins when healthcare professionals capture OV-RDT test images through Android and iOS mobile applications. These images are immediately transmitted to Azure Cloud Storage, the secure initial repository for raw diagnostic data. The architecture employs a Service Bus to manage asynchronous communication between system components, directing these images to the AI Server comprised of two critical subsystems: image quality control and the EffNet-B5 R-powered grading model, which classifies infection severity into five levels (0, +1, +2, +3, +4). The backend data integration process, illustrated in Fig. 7, shows how the AI-processed results are structured within a sophisticated relational database schema. The system combines three primary data sources: OV screening results with patient information from the mobile application (stored in the OV_results table with a RowKey primary key), healthcare service center data (Health_centers table), and geographical coordinates (citizeninfo_health table). Through table-to-table transformations and joinings using Hospital_ID as a foreign key, the system enriches the diagnostic data with demographic information (patient titles, ages) and creates derived fields like age_intervals and OS_system. For data cleansing, four issues are concerned: data standardization, data range validation, duplicate removal, and data consolidation. The final integrated table is a comprehensive data warehouse that powers the Google Looker Studio-based intelligent dashboard (https://cloud.google.com/looker-studio), enabling epidemiological analysis with complete geographical context.

### Assessment results

The usability assessment of the OV-RDT mobile application revealed exceptional user satisfaction with an overall mean score of 4.41 (”Extremely Satisfied”), as shown in Table 4, with particularly high ratings for interface design (4.82) and ease of use (4.76). This satisfaction level exceeds even that of the physical OV-Rapid test kit, which scored 4.36 overall in Table 3, where users particularly valued the test’s result display duration (4.59) and user-friendly design (4.45) while finding the grading colour chart somewhat less intuitive (3.50) compared to other aspects. From the 39 respondents (78% response rate), 17 mobile application users demonstrated strong confidence in the AI-powered features, especially appreciating the system’s ability to expedite result processing (4.47). However, AI interpretation accuracy scored slightly lower (3.88).

The application’s potential for broader implementation is evidenced in Table 5, which reveals overwhelming user confidence in the technology’s future utility—52.9% of respondents indicated they would ”Definitely” use the application in other campaigns and activities. In comparison, an additional 41.2% reported they would ”Most Likely” do so, resulting in a combined positive adoption intention of 94.1%. Notably, not a single respondent selected the ”Probably not” or ”Not” options, underscoring the perceived value and reliability of the AI-assisted diagnostic approach. Despite the overwhelmingly positive reception, respondents provided valuable suggestions for enhancement, including improved image stabilization through tripod support, implementation of familiar camera button functionality, enhanced application stability, and visual positioning guidance.

**Table 3:** Summary scores representing the user satisfaction on the OV-Rapid test kit.

Topics	$\bar{X}$	Satisfaction level
The OV-Rapid test kit is user-friendly.	4.45	Extremely satisfied
Duration for displaying the OV-Rapid test kit results.	4.59	Extremely satisfied
The results are easy to comprehend and analyze.	3.91	Very satisfied
The grading color chart for infection levels is readily distinguishable.	3.50	Very satisfied
**Overall satisfaction**	**4.36**	**Extremely satisfied**

**Table 4:** Summary scores representing the user satisfaction on the OV-RDT mobile application.

Topics	$\bar{X}$	Satisfaction level
The OV-RDT mobile application was easy to use.	4.76	Extremely satisfied
The app’s interface was user-friendly and intuitive.	4.82	Extremely satisfied
Positioning to capture images of the OV-Rapid test kit is convenient.	4.35	Extremely satisfied
The AI system accurately interprets results.	3.88	Very satisfied
The AI system significantly speeds up the process of reading and recording results.	4.47	Extremely satisfied
**Overall satisfaction**	**4.41**	**Extremely satisfied**

**Table 5:** User willingness to use of the OV-RDT mobile application in other campaigns and activities.

Item	N	%
Definitely	9	52.90
Most likely	7	41.20
Unsure	1	5.90
Probably not	0	0.00
Not	0	0.00

## Dashboard

The proposed dashboard was developed and designed to meet the stakeholders’ requirements through a systematic interviewing process. From this process, we identified the key information needed: the number of healthy individuals, infected individuals across levels $1-4$, app users on iOS and Android platforms, and female and male participants. The preprocessed data is imported into the server using Google Spreadsheets via API to create a data warehouse encompassing relevant dimensions, fields, and tables, all utilized to generate various views. The data is then sliced using filters and parameters stored in the extracted data source and visualized through automated dashboards in Google Looker Studio. This structured approach enables clear, insightful visual representations of the screening campaign’s progress and outcomes.

An automated intelligent dashboard has been developed to support spatial strategic management by analyzing information obtained via the OV-RDT mobile application. We have developed three interactive dashboards: Overview Metrics, Geospatial Visualization, and the OV-RDT mobile application insight. Developing three distinct types of dashboards enables a more versatile and tailored data analysis and presentation approach. Each dashboard serves different purposes or target audiences, providing unique features and functionalities to meet specific needs. This diversity in dashboard options enhances the overall usability and effectiveness of the automated report generation, supporting a spatial strategic management system more efficiently. The data presented on the interactive dashboards in this work encompasses OV screening results from February 15, 2022, to March 20, 2024. The details of each type of dashboard are outlined as follows:

### Overview metrics

**Fig. 8:**
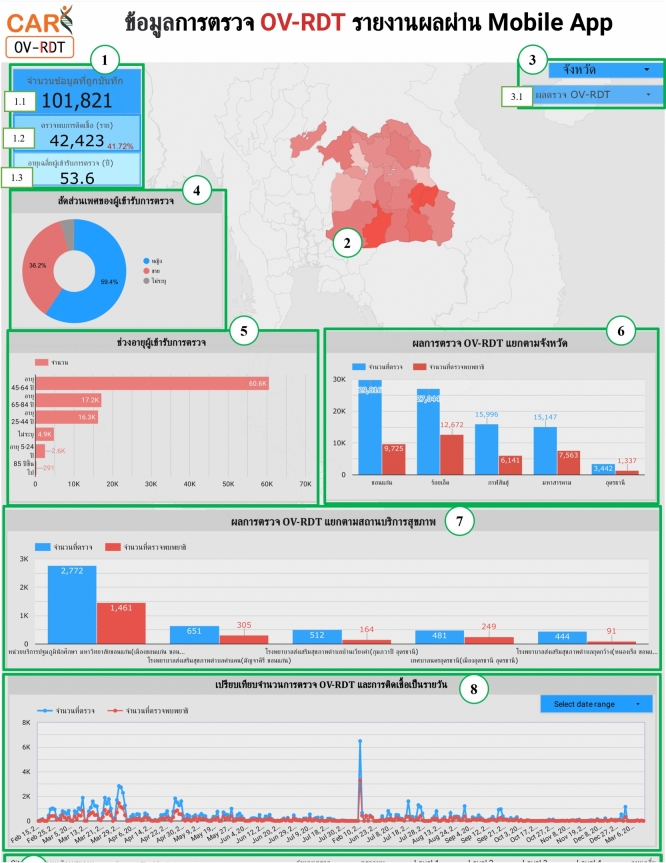
Nine parts of the overview metrics page of the data analytic dashboard including of (1) statistical values of (1.1) number of the total participants, (1.2) number of infected participants, and (1.3) the average age of the total participants, (2) infection map with coloring based on the number of infected ones, (3) dropdown list of selected province and (3.1) specified infection status, (4) donut chart for portion of the specified and not specified sexes, (5) bar chart for age intervals of the total participants, (6) bar chart for total participants v.s. infected ones on the top five numbers of total participants, (7) bar chart for total participants v.s. infected ones on the top five number of total participants in healthcare service centres, (8) line chart for number of total v.s. infected participants on the specified time range and (9) statistical table of the total participants, portion of infection, infection level and the average age on each healthcare service centre. The map in the figure is originally generated by the authors through https://cloud.google.com/looker-studio.

The metrics analysis dashboard aims to show the overall statistical value of the mass opisthorchiasis screening campaign. The Overview Metrics Dashboard, illustrated in Fig. 8, is described by the following corresponding numbers:

- Number 1: This represents a scorecard that displays statistical information related to the total OV-RDT data recorded via the OV-RDT mobile application, which is compatible with both Android and iOS operating systems. The scorecard includes (1.1) total participants, (1.2) the number of infected participants, and (1.3) the average age of participants. The health facilities’ code (hopital_ID) can be utilized to search for health services places.
- Number 2: This section presents a Geo chart illustrating the percentage of liver fluke infection. The result is formatted as a percentage and displayed on a province-segmented map. The intensity of the colors on the map reflects the density of screening participants: darker colors indicate a higher number of infections in a given province, while lighter colors suggest a relatively lower number. This map provides a visual representation of the distribution of infection efforts across provinces, enabling an assessment of regional variations in infection rates for liver fluke screening.
- Number 3: This refers to a drop-down menu that allows users to control and filter results based on specific provinces using the province field as a control field.
- Number 4: This refers to a pie chart displaying the percentage distribution of gender among all participants, highlighting the relative proportions of males, females, and unknowns in the screening.
- Number 5: This represents a bar chart illustrating the number of participants in the screening, categorized by age group.
- Number 6: This represents a column chart comparing the number of screening participants to the number of individuals diagnosed with liver fluke. The chart is focused on the top five provinces with the highest participant numbers.
- Number 7: This represents a column chart that compares the number of screening participants to the number of individuals diagnosed with liver fluke. The data is categorized by the top five health facilities with the most participants.
- Number 8: This represents a line chart displaying the number of screening participants, and the number of individuals diagnosed with liver flukes over time, starting from the commencement of OV-RDT data recording via the OV-RDT mobile application to the present. Additionally, data range control properties are added to allow users to set the date range, enabling the display of data specific to the period of interest.
- Number 9: This section displays a table providing information on health service centres, sorted in descending order based on the number of participants in the screening. It includes the percentage of people who tested positive for liver flukes, the severity levels (from level 1 to level 4), the average age of participants, and the number of participants categorized by gender.

### Geospatial visualization

**Fig. 9:**
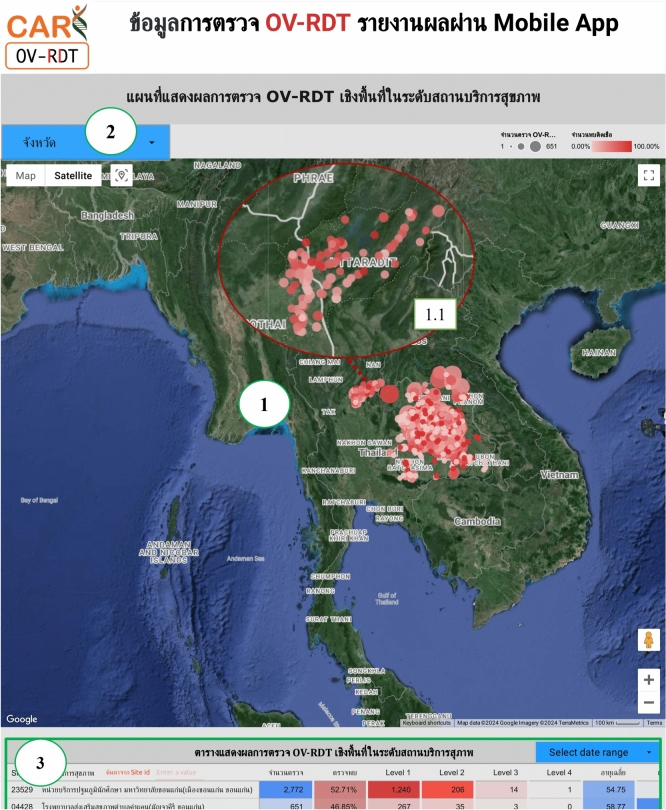
Three parts of geospatial page of the data analytic dashboard including of (1) geospatial map various size of circles colored by portion of the infection, (1.1) enlarge image at the specified area, (2) dropdown list of the provinces, and statistical table of the total participants, portion of infection, infection level and the average age on each healthcare service centre. All maps in the figure are originally generated by the authors through https://cloud.google.com/looker-studio. The satellite image is from Google Imagery.

The geospatial visualization dashboard is designed to display the screening locations of the mass opisthorchiasis screening campaign using interactive maps. The following numbers correspond to the features shown in Fig. 9:

- Number 1: This geospatial visualization utilizes interactive maps to present the distribution of liver fluke infections across different health service centres at the sub-district level, with larger circles representing more participants at each health service centre and smaller circles indicating fewer participants. The map uses color intensity to represent infection rates, with darker colors indicating a higher percentage of infections and lighter colors suggesting a relatively lower rate of infections and (1.1) The map allows users to zoom in and enlarge the image within a specified area of interest, providing a more detailed view of the selected region.
- Number 2: This refers to a drop-down menu that allows users to control and filter results based on specific provinces
- Number 3: This is a tabular representation of OV screening at health service centres at the sub-district level. The table lists health service centres in descending order based on the number of individuals participating in the screening. Each row in the table includes the number of participants who tested positive for liver fluke, categorized into different severity levels (from level 1 to level 4). The table also provides the average age of the participants and the number of inspectors divided by gender. Additionally, users can search for specific health service centres within the table by entering the health service centre code.

### OV-RDT application insight

**Fig. 10:**
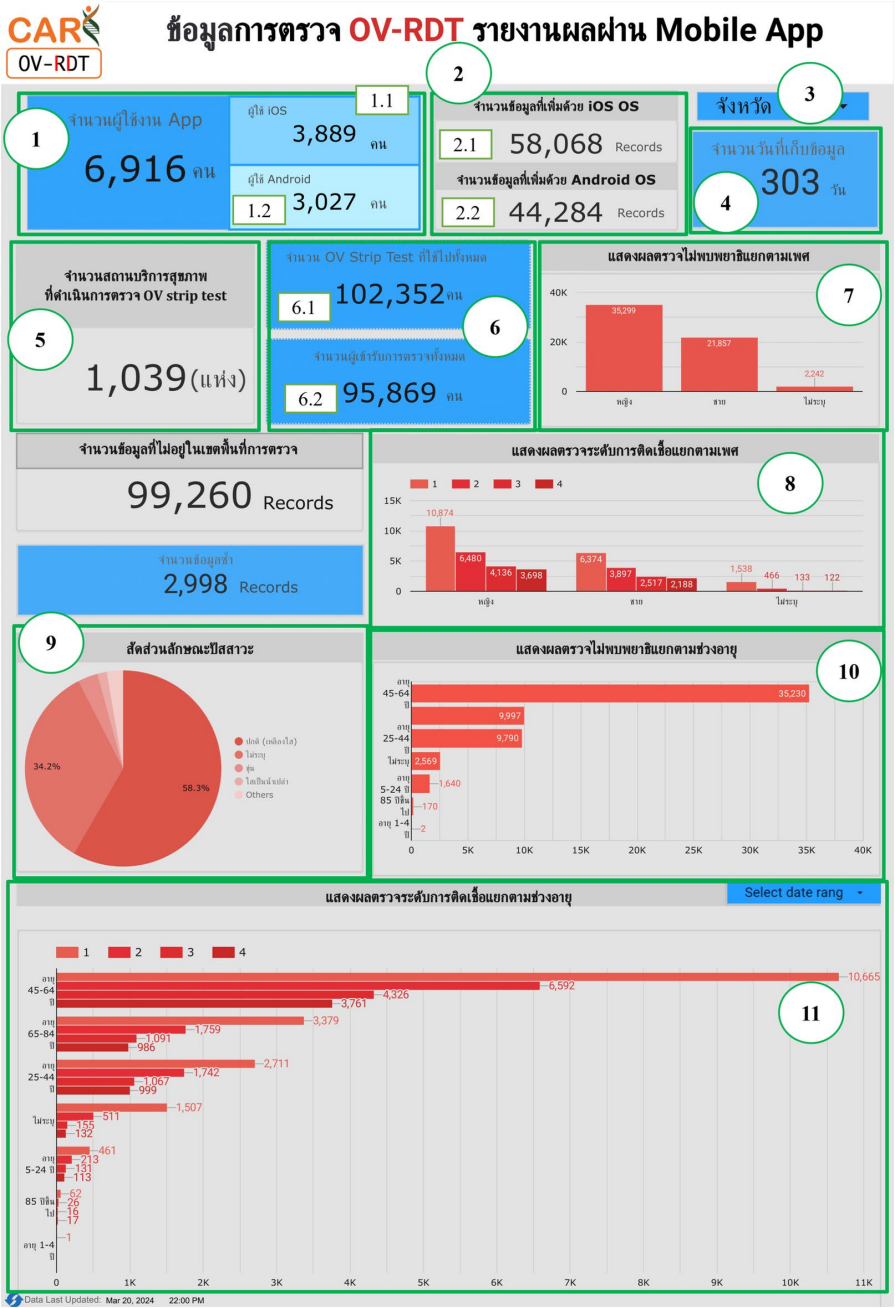
The OV-RDT insight page of the data analytic dashboard for monitoring mobile application usage including of (1) the number of total mobile application users divided into (1.1) IOS users and (1.2) Android users, (2) the numbers of collected records from IOS and Android applications, (3) the selected province, (4) the number of days, (5) the number of healthcare service centre, (6.1) the number of OV Strip used tests, (6.2) number of participants, (7) the number of normal participants in specified and not specified sexes, (8) the number of strip test results, (9) the pie chart of the portion of five type of urine aspects, (10) the bar chart of the numbers of normal participants divided by five age intervals, and (11) the bar chart of the numbers of infected participants characterized by five age intervals and four levels of infection.

The OV-RDT dashboard designed specifically for developers, also known as “App Insight”, focuses on providing detailed insights and analytics related to the performance and usage of the OV-RDT mobile application. Figure 10 shows key components of this dashboard including:

- Number 1: This is a scorecard that displays statistics on the number of OV-RDT mobile application users across Android and iOS operating systems: (1.1) the number of OV-RDT mobile application users on iOS operating systems and (1.2) the number of OV-RDT mobile application users on Android operating systems.
- Number2: This is a scorecard showcasing statistics on the number of OV screening data entries recorded through the OV-RDT mobile application on both Android and iOS operating systems, (2.1) the number of collected records from iOS, and (2.2) the number of collected records from Android.
- Number3: This refers to a drop-down menu that allows users to control and filter results based on specific provinces.
- Number 4: The scorecard, presents statistics regarding the number of days taken to collect data on OV screenings.
- Number 5: The statistical data presented on the scorecard, indicates the aggregate number of health service centres that have implemented the screening procedure.
- Number 6: The scorecard illustrating statistical data on the aggregate quantity of OV-Rapid test kits utilized for screening and the total number of participants across all areas is compiled, (6.1) the total number of OV-Rapid test kits utilized for screening across all areas, and (6.2) the total number of participants across all areas.
- Number 7: The column chart presents data on the number of negative screening results, stratified by gender.
- Number 8: The bar chart illustrates the number of participants who tested positive, exhibiting infection levels 1-4, categorized by gender. The gender categories are arranged in descending order based on the number of participants at each infection level.
- Number 9: The pie chart illustrates the distribution of the five most prevalent urine characteristics identified during the screening process.
- Number 10: A bar chart depicting the number of participants who tested positive, comparing severity levels categorized by age range. The age ranges are arranged in descending order based on the number of participants who tested negative.
- Number 11: A bar chart illustrating the number of participants infected with liver fluke, comparing severity levels categorized by age range. The age ranges are arranged in descending order based on the number of participants who tested positive.

## OV-RDT platform

This section provides an overview of the architectural framework of the platform, highlighting the workflow involved in capturing, processing, and analyzing data from the OV-Rapid test kit. Following this, we will present the current state of the screening campaigns, detailing their implementation in northeastern Thailand.

### Architecture

The OV-RDT platform supports Android and iOS mobile applications through a system architecture comprising three components: AI server, data processing server, and intelligent dashboard. As shown in Fig. 1, the workflow begins when users capture OV-Rapid test kit images using the camera template in the OV-RDT mobile application. These test kit images are uploaded to the cloud storage, with processing tasks queued in the service bus. The AI server then processes tasks through two subsystems: Image Quality Control, which filters images to meet quality standards, and OV-RDT-Grading, which analyzes and grades filtered images. The system generates grading reports and collects patient information (patient_ID, hospital_ID, user_ID) for storage in the cloud database. Subsequently, the data processing server extracts information from the cloud database for presentation. The intelligent dashboard functions as a monitoring and analysis tool for healthcare professionals. This architecture facilitates mobile diagnostic testing while ensuring data quality, storage, and visualization for clinical decision-making.

### Current state

**Fig. 11:**
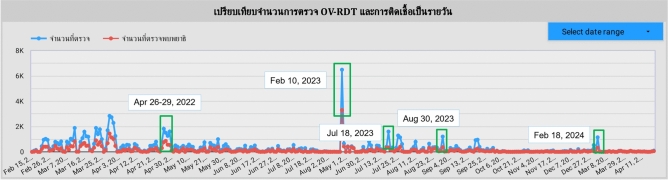
A timeline shows the number of input data submitted to the system each day. Blue color represents the number of the tests and red color represents the number of OV infected. Green boxes highlight four mass screening campaigns described in the opisthorchiasis screening section.

This section delineates five principal campaigns, each characterized by substantial participation within Thailand’s northeastern region. The campaign chronology for 2022 encompasses a single campaign, while 2023 features three distinct campaigns. The year 2024 includes one campaign. This temporal distribution is visually represented in the line chart presented on our dashboard, as depicted in Fig. 11. In a mass screening campaign for OV in Thailand, a total of 101, 821 individuals participated, and 42, 423 (41.70%) tested positive for infection. The average age of participants was 53.60 years. The gender distribution was 60, 487 (59.40%) females, 36, 833 (36.20%) males, and 4, 874 (4.80%) of unknown gender. Most participants, totaling 60, 574 individuals (59.50%), were between 45 and 64 years old, as shown in Table 6.

**Table 6:** The OV-RDT platform background characteristics of study participants, data represent no. (%) of participants.

Characteristic	Participants, No. (%)
Sex	
Female	60,487 (59.40%)
Male	36,833 (36.20%)
Unknown	4,501 (4.40%)
Age (mean = 53.6 years)	
5-24	2,558 (2.50%)
25-44	16,309 (16.00%)
45-64	60,574 (59.50%)
65-84	17,212 (16.90%)
$\ge 85$	291 (0.30%)
Unknown	4,874 (4.80%)

**Fig. 12:**
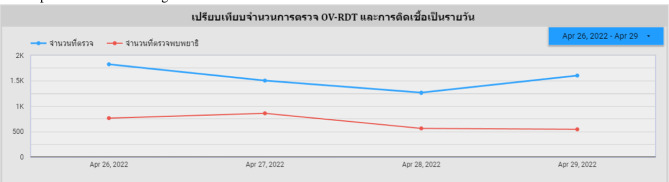
A timeline shows the number of input data on the day of mass screening for opisthorchiasis for campaign 1, which is four days.

### Mass screening campaign

**Fig. 13:**
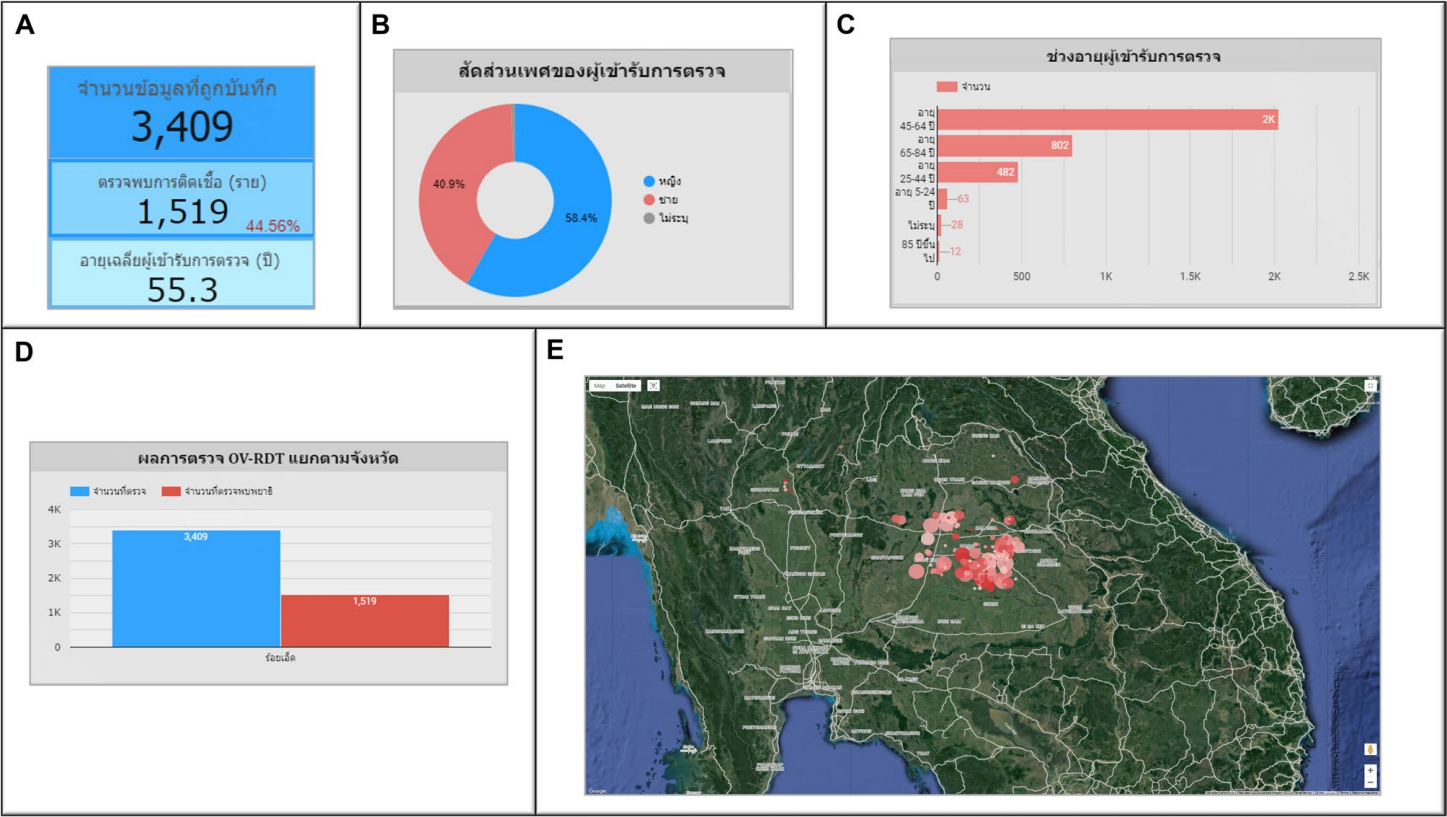
Analysis of mass screening for opisthorchiasis for campaign between April 26-29, 2022. The map in the figure is originally generated by the authors through https://cloud.google.com/looker-studio. The satellite image is from Google Imagery.

**Use case 1: Mass screening campaign between April 26–29, 2022** The event was conducted in Roi-Et Province over four consecutive days. Figure 12 illustrates the temporal progression of participant attendance and infection rates throughout the event, with a total attendance of 3,409 participants. Among these attendees, 1,519 individuals (44.56%) were confirmed positive for infection, participants with a mean age of 55.30 years, as depicted in Fig. 13A. An analysis of the gender distribution among screened participants revealed that 58.40% were female, 40.90% were male, and 0.70% were unidentified, as illustrated in Fig. 13B. Most participants, comprising 2,022 individuals (59.30%), fell within the age range of 45 to 64 years, as demonstrated in Fig. 13C. Notably, this campaign exclusively involved citizens from Roi-Et province, as evidenced in Fig. 13D. The geospatial representation presented in Figure 13E indicates that Selaphum Hospital, located in Selaphum District, Roi Et Province, exhibited the highest level of participation in the screening process, as denoted by the largest circle size. Furthermore, Ban Nong Sim Subdistrict health-promoting hospital, situated in Kaset Wisai District, Roi-Et Province, recorded the highest proportion of infected individuals, with an infection rate of 95.83%, represented by the most intensely red-colored circle on the map.

**Fig. 14:**
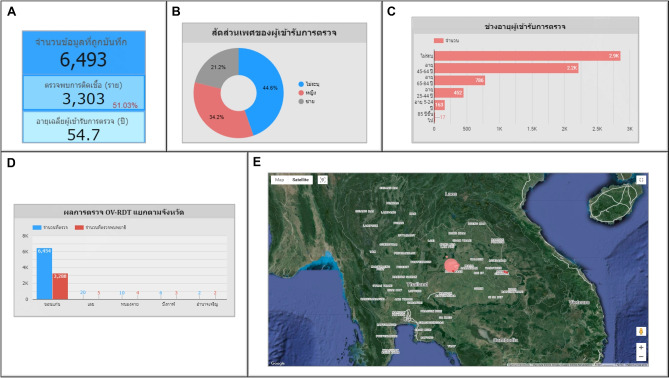
Analysis of mass screening for opisthorchiasis for campaign on February 10, 2023. The map in the figure is originally generated by the authors through https://cloud.google.com/looker-studio. The satellite image is from Google Imagery.

**Use case 2: Mass screening campaign on February 10, 2023** This event represents the largest campaign, coinciding with World Cholangiocarcinoma Day (World CCA Day) 2023 at Khon Kaen University in Khon Kaen, Thailand. A total of 6,493 participated in the screening process. Among them, 3,303 individuals, accounting for 51.03%, tested positive for infection. The average age of the participants was 54.70 years, as shown in Fig. 14A. The participants were females 2,219 persons (34.20%), males 1,375 persons (21.20%), and 2,899 persons (44.60%) of unspecified genders, as depicted in Fig. 14B. Most participants were between the ages of 45 and 64, a total of 2,216 persons (34.10%), as illustrated in Fig. 14C. Participants came from five nearby provinces: Khon Kaen, Loei, Nong Khai, Bueng Kan, and Amnat Charoen, as in Fig. 14D. Figure 14E depicts the screening results conducted by health service centres on a geospatial map. The Student Health centre at Khon Kaen University in Mueang Khon Kaen District, Khon Kaen Province, exhibited the highest participation rate, represented by the largest circle size. Furthermore, Khok Samran Sub-district health-promoting hospital in Ban Haed District, Khon Kaen Province, demonstrated the highest proportion of infected individuals at 67.92%, represented by the most intense red color on the map.

**Fig. 15:**
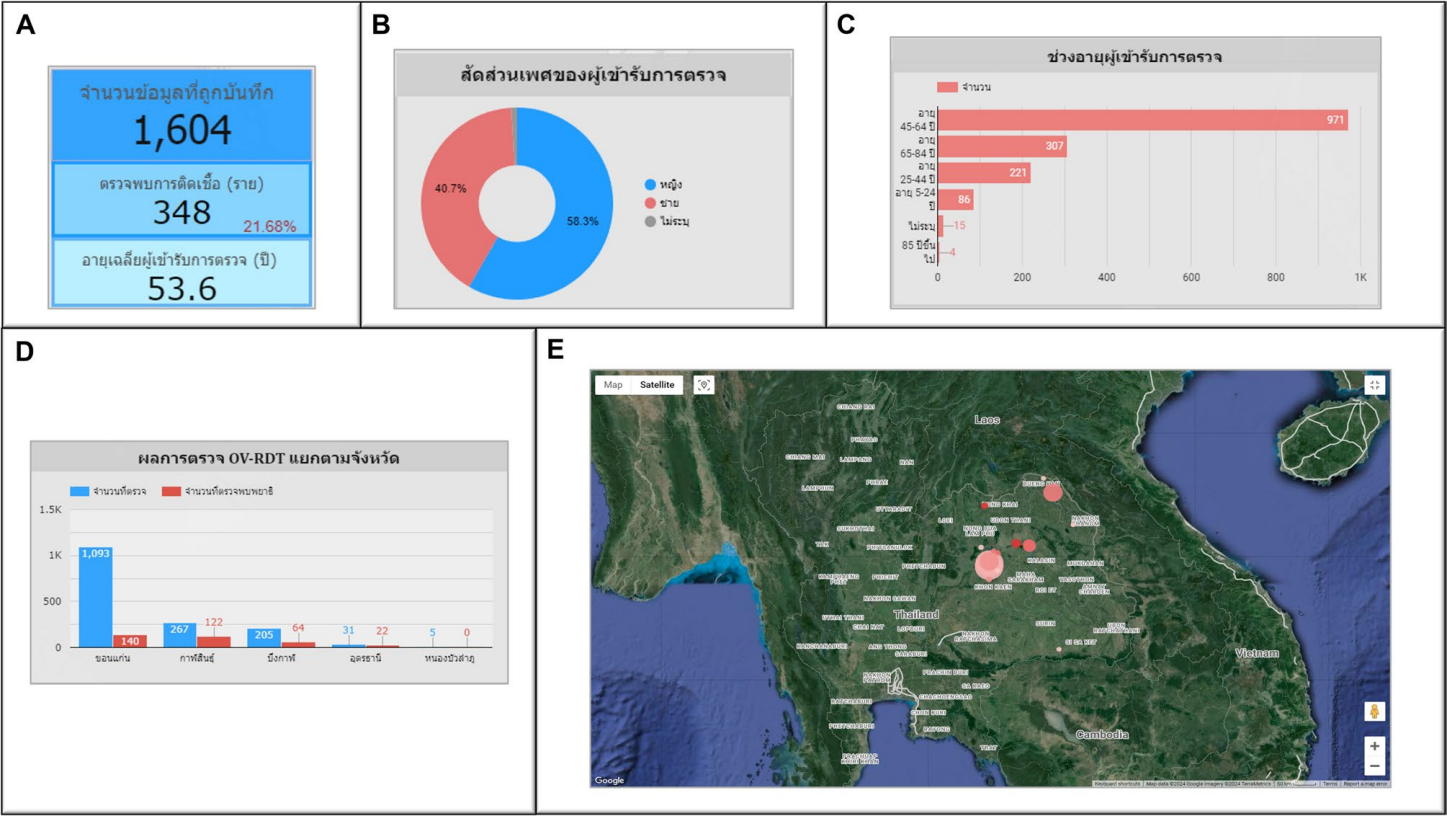
Analysis of mass screening for opisthorchiasis for campaign on July 18, 2023. The map in the figure is originally generated by the authors through https://cloud.google.com/looker-studio. The satellite image is from Google Imagery.

**Use case 3: Mass screening campaign on July 18, 2023** In the Khon Kaen province, the number of participants was 1,604 persons. Among them, 348 persons (21.68%) tested positive for infection, with an average age of 53.60 years, as shown in Fig. 15A. The gender breakdown of those tested was 58.30% female, 40.70% male, and 1.00% unknown, as illustrated in Fig. 15B. The participants mainly consisted of people between 45 and 64 years of age, totaling 971 persons (60.50%), shown in Fig. 15C. Khon Kaen province had the highest number of participants, followed by Kalasin, Bueng Kan, Udon Thani, and Nong Bua Lamphu, respectively, as shown in Fig. 15D. In the geospatial map illustrated in Fig. 15E, Yang Kham Sub-district health-promoting hospital in Nong Ruea District, Khon Kaen Province, demonstrated the highest participation in the screening (largest circle size), and Ban Kho Sub-district health-promoting hospital in Ban Phue District, Udon Thani Province, exhibited the highest proportion of infected individuals at 70.97% (the circle is the reddest).

**Fig. 16:**
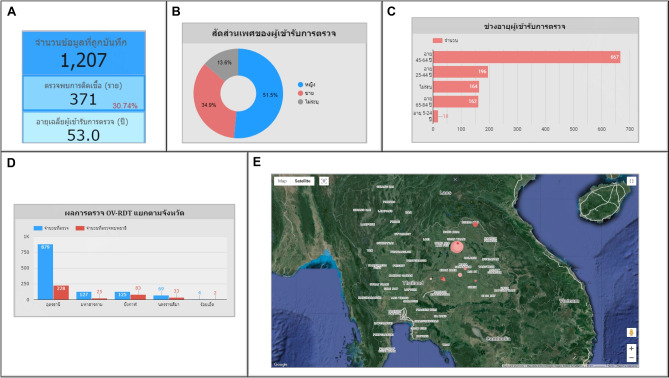
Analysis of mass screening for opisthorchiasis for campaign 4 on August 30, 2023. The map in the figure is originally generated by the authors through https://cloud.google.com/looker-studio. The satellite image is from Google Imagery.

**Use case 4: Mass screening campaign on August 30, 2023** In Udon Thani province, out of the 1,207 persons screened, 371 persons (30.74%) tested positive for infection, with an average age of 53, as illustrated in Fig. 16A. The gender distribution revealed 51.50% female, 34.90% male, and 13.60% unknown, as shown in Fig. 16B. Most participants were between 45 and 64 years old, totaling 667 persons (55.30%), as illustrated in Fig. 16C. Udon Thani province recorded the highest participation, with neighboring provinces including Maha Sarakham, Bueng Kan, Nakhon Ratchasima, and Roi Et, respectively, as shown in Fig. 16D. In the geospatial map presented in Fig. 16E, Ban Wiang Kham Sub-district health-promoting hospital in Kumphawapi District, Udon Thani Province, exhibited the highest screening participation (largest circle size). Ban Na Kham Khaen Sub-district health-promoting hospital in Si Wilai District, Bueng Kan Province, demonstrated the highest proportion of infected individuals at 66.40% (the circle is the reddest).

**Fig. 17:**
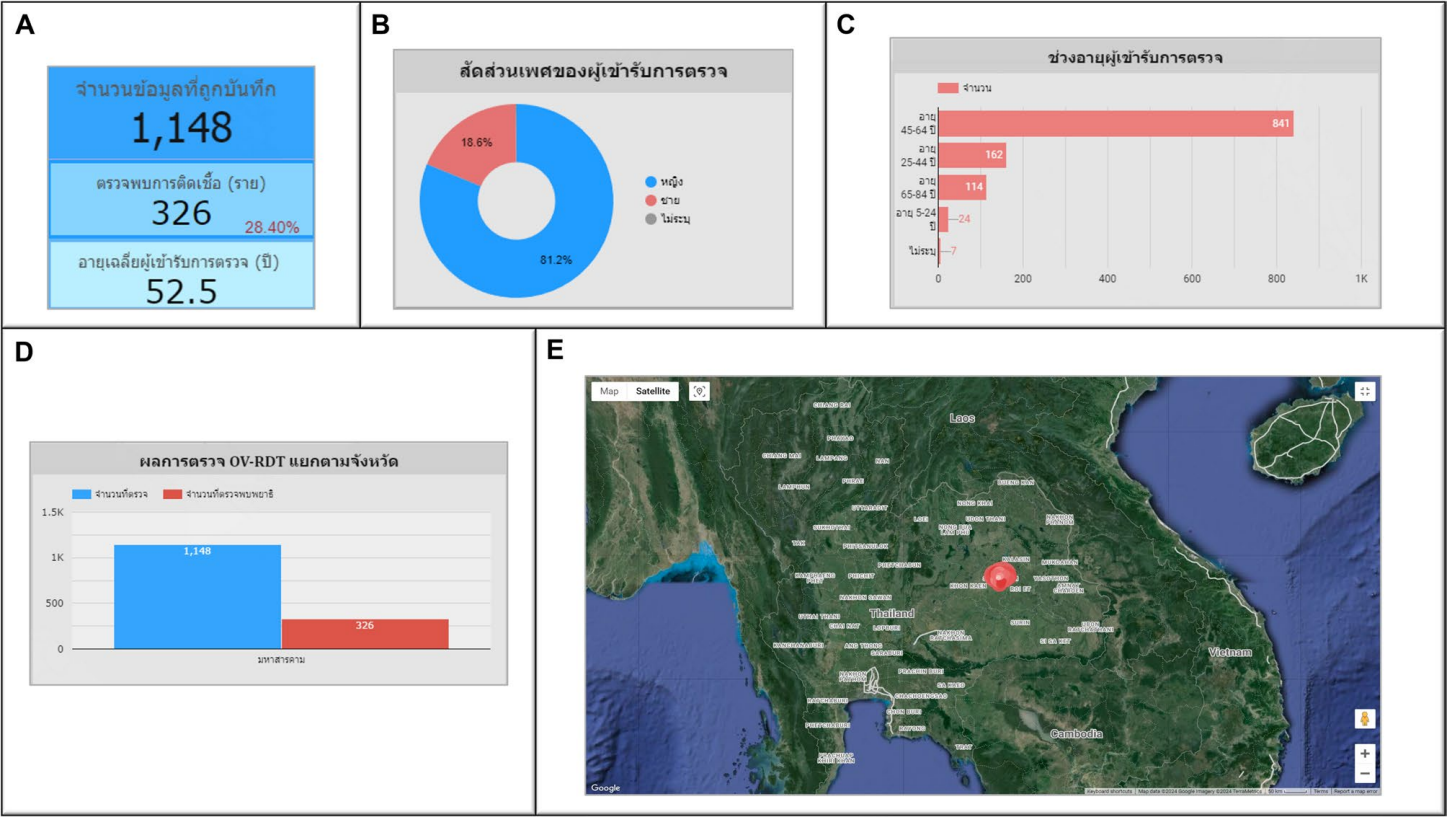
Analysis of mass screening for opisthorchiasis for campaign 5 on February 18, 2024. The map in the figure is originally generated by the authors through https://cloud.google.com/looker-studio. The satellite image is from Google Imagery.

**Use case 5: Mass screening campaign on February 18, 2024** This event was held in Maha Sarakham Province, where 1,148 participated in the screening. Among them, 326 persons (28.40%) tested positive for infection, with an average age of 52.50 years, as shown in Fig. 17A. The gender ratio of those screened indicated that 81.20% were female, 18.60% were male, and 0.20% could not be identified, as shown in Fig. 17B. Most participants, totaling 841 persons (73.30%), were between 45 and 64, as shown in Fig. 17C. This campaign has participants from citizens only in Maha Sarakham province, as illustrated in Fig. 17D. The geospatial map shown in Fig. 17E, Ban Nong Waeng Sub-district health-promoting hospital in Mueang Maha Sarakham District exhibited the highest participation in the screening (largest circle size). Uthai Thani Community Medical centre in Mueang Maha Sarakham District displayed the highest proportion of infected individuals, at 50.00% (the circle is the reddest).

Thus, the Opisthorchiasis screening campaign is a national initiative aimed at raising awareness about Cholangiocarcinoma (CCA), a severe cancer affecting the bile ducts both inside and outside the liver. This poorly understood disease is increasing in incidence globally. CCA causes several vague symptoms, making it very difficult to diagnose. In some cases, misdiagnosis with other less serious conditions leads to misdiagnosis before receiving the correct diagnosis. After a lengthy and challenging diagnostic process, patients often encounter a disease they are unfamiliar with, which typically has a poor prognosis and limited treatment options. This campaign aims to facilitate a more straightforward diagnostic journey for individuals affected by cholangiocarcinoma, ensuring the process is timely, decisive, and well-supported.

## Discussion and conclusion

We introduced the OV-RDT platform, a novel system integrating deep learning, cloud computing, and mobile technology for mass opisthorchiasis screening specifically designed for northeastern Thailand, where the disease burden is most severe. The platform’s core EfficientNet-B5-based deep learning architecture achieved 95.00% accuracy in OV infection detection and 98.00% accuracy in image quality assessment within this endemic region. This robust performance enabled large-scale deployment, successfully processing over 100,000 samples across northeastern Thailand.

The distributed architecture effectively addresses scalability challenges through a cloud-based infrastructure supporting real-time data processing and cross-platform mobile applications. The system’s intelligent dashboard provides comprehensive analytics and geospatial visualization capabilities, facilitating evidence-based public health decision-making. Field validation demonstrated significant clinical utility, with healthcare professionals reporting high satisfaction rates (4.41/5.00) in real-world deployments. It is important to note that our platform was developed and validated exclusively in northeastern Thailand, where opisthorchiasis prevalence is exceptionally high and represents a major public health challenge. The unique epidemiological characteristics of this region, including dietary habits and environmental factors, may limit the direct generalizability of our findings to other geographical areas. External validation studies in different endemic regions would be essential before broader deployment of the platform.

While the binary classification task (negative vs. positive) achieved excellent performance (95% accuracy), the multi-class grading task presented greater challenges, achieving 66% accuracy with a Cohen’s Kappa of 0.55, indicating moderate agreement. This performance gap reflects the inherent subjectivity in visual assessment of T-band color intensity, where even expert raters may disagree on borderline cases between adjacent grades (e.g., Level 2 vs. Level 3).

To address these limitations in clinical practice, our platform incorporates two key mitigation strategies:

First, the AI grading suggestions are implemented as default values that healthcare workers can review and modify based on their clinical expertise. This human-in-the-loop design ensures that the AI serves as a decision support tool rather than an autonomous diagnostic system, particularly important for borderline cases where grading uncertainty is highest.

Second, the platform implements server-side threshold adjustment capabilities that allow for dynamic calibration based on batch-specific characteristics or epidemiological requirements. For example, if a particular batch of test strips exhibits systematic variations in color development, thresholds can be adjusted centrally to maintain diagnostic accuracy. This flexibility is particularly valuable in resource-limited settings where test strip quality may vary due to storage conditions or manufacturing differences.

Future research directions should focus on enhancing the system’s distributed computing capabilities, particularly offline functionality for remote areas and integration with existing healthcare information systems. Long-term clinical validation studies are essential to quantify the platform’s impact on early detection rates and treatment outcomes. The expansion of the system throughout Southeast Asia and the Lower Mekong Basin countries presents opportunities for standardizing cross-border screening protocols and implementing advanced security measures through blockchain technology.

Additionally, while the current dashboard visualization provides valuable insights, future iterations should better highlight regional epidemiological trends to support more targeted interventions. Incorporating spatiotemporal analysis that examines infection trends over time and potential seasonal variation effects would make findings more actionable for public health authorities. Furthermore, developing predictive analytics capabilities using AI to forecast future outbreaks based on environmental, demographic, and behavioral data represents a promising direction that could transform the platform from a diagnostic tool to a preventive one. Such forecasting models could leverage the extensive dataset being accumulated to identify high-risk areas before outbreaks occur. Future technical enhancements should also explore image preprocessing techniques such as color normalization and stain standardization, which could reduce the impact of lighting variations and device-specific color rendition on model performance. Such preprocessing methods, successfully applied in digital pathology and other medical imaging domains, may improve the system’s ability to consistently grade T-band intensities across diverse field conditions. Sourcing additional image datasets that are representative of other endemic regions beyond northeastern Thailand, training and validating the generalization performance of the EfficientNet-B5-based model is essential during this expansion. Given that our current validation is limited to northeastern Thailand—the region with the most severe disease burden—comprehensive external validation studies in diverse populations and geographical settings are crucial for establishing the platform’s broader applicability.

The OV-RDT platform demonstrates the potential of AI-driven solutions in public health screening, particularly in standardizing diagnostic processes and eliminating subjective interpretation in immunochromatographic tests. This standardization, combined with automated data collection and analysis, represents a significant advancement in opisthorchiasis screening methodology. The platform’s success in reducing diagnostic variability while maintaining high accuracy could serve as a model for applying machine learning to other neglected tropical diseases.

Continued advancement requires interdisciplinary collaboration between medical professionals, computer scientists, and public health researchers. This cooperation will be crucial in evolving the platform’s capabilities while ensuring its practical application in resource-limited settings, ultimately working toward more effective disease control in endemic regions.The GradCAM analysis presented in this study addresses a critical need for interpretability in medical AI systems. By demonstrating that our model focuses on clinically relevant regions (the T-band), we provide healthcare workers with visual explanations that can enhance trust and facilitate adoption of the technology. This interpretability is particularly important in resource-limited settings where AI systems must be transparent to gain acceptance from medical professionals. The incorporation of GradCAM visualization confirms that our deep learning model makes decisions based on clinically relevant features, providing the transparency necessary for deployment in real-world healthcare settings.

### Limitations and clinical implementation considerations

The moderate performance in multi-class grading (66% accuracy) compared to binary classification (95% accuracy) represents a key limitation that must be considered in clinical deployment. This performance gap primarily stems from the subjective nature of visual intensity grading, where inter-rater variability exists even among experts. However, our platform’s design specifically addresses this limitation through:

- 1. Human-in-the-loop verification, where AI suggestions serve as initial assessments that healthcare workers can validate or adjust based on clinical context
- 2. Adaptive threshold management at the server level, enabling real-time calibration based on batch-specific variations or changing epidemiological requirements
- 3. Continuous model improvement through accumulation of expert-verified labels from field deployments

Future work should focus on collecting larger datasets with multiple expert annotations to better capture the inherent variability in visual grading, potentially implementing ensemble methods or uncertainty quantification to flag cases requiring additional human review. Additionally, investigating alternative biomarkers or test formats that provide more objective quantification could further enhance the reliability of infection intensity assessment.

## Supplementary Information

Supplementary Information.

## Data Availability

The datasets used and/or analysed during the current study available from the corresponding author on reasonable request.
