# Supplementary material for: AI-enhanced rapid diagnostic testing platform for mass opisthorchiasis screening
Source: Sci Rep. 2025 Aug 23;15:31062. doi: 10.1038/s41598-025-16893-7 (PMC12374986; doi:10.1038/s41598-025-16893-7)
Supplement: Supplementary file 1 — Supplementary Information. [file 41598_2025_16893_MOESM1_ESM.pdf]

OV-RDT Supplementary

Teble S1: Cohen's Kappa scores for OV-RDT grading and status across eight machine learning models.

**Reliability measurement by Cohen Kappa score**		
	Cohen Kappa	
Method	OV-RDT grading	OV-RDT status
EffNet-B5 R	0.55	0.83
EffNet-B5	0.53	0.82
SVM	0.37	0.49
RF	0.29	0.34
ResNet50 R	0.47	0.68
ResNet50	0.47	0.69
MobileNetV2 R	0.35	0.64
MobileNetV2	0.43	0.71

Table S2: Tukey's HSD post-hoc analysis comparing model performance in estimating OV infectious level grading and status.

**Classify**	**Models**	**Performance**			
		**Accuracy**	**Recall**	**Precision**	**F1-score**
OV-RDT grading (0, +1, +2, +3, +4)	**EffNet-B5 R**	**0.66 ± 0.06**^{a}	**0.66 ± 0.06**^{a}	**0.68 ± 0.06**^{a}	**0.66 ± 0.06**^{a}
	EffNet-B5	0.63 ± 0.06^{a,b}	0.63 ± 0.06^{a,b}	0.64 ± 0.06^{a,b}	0.62 ± 0.06^{a,b}
	SVM	0.48 ± 0.07^{d}	0.48 ± 0.07^{d}	0.50 ± 0.06^{c}	0.48 ± 0.07^{d}
	RF	0.44 ± 0.06^{d}	0.44 ± 0.06^{d}	0.46 ± 0.07^{c}	0.44 ± 0.06^{d}
	ResNet50 R	0.58 ± 0.07^{b,c}	0.58 ± 0.07^{b,c}	0.61 ± 0.08^{b}	0.58 ± 0.07^{bc}
	ResNet50	0.58 ± 0.07^{b,c}	0.58 ± 0.07^{b,c}	0.57 ± 0.09^{b}	0.57 ± 0.07^{b,c}
	MobileNetV2 R	0.55 ± 0.06^{c}	0.55 ± 0.06^{c}	0.59 ± 0.06^{b}	0.55 ± 0.06^{c}
	MobileNetV2	0.48 ± 0.05^{d}	0.48 ± 0.05^{d}	0.49 ± 0.06^{c}	0.46 ± 0.05^{d}
OV-RDT Status (Negative (0), Positive (1–4))	**EffNet-B5 R**	**0.95 ± 0.03**^{a}	**0.95 ± 0.03**^{a}	**0.96 ± 0.03**^{a}	**0.95 ± 0.03**^{a}
	EffNet-B5	**0.95 ± 0.03**^{a}	**0.95 ± 0.03**^{a}	**0.95 ± 0.03**^{a}	**0.95 ± 0.03**^{a}
	SVM	0.84 ± 0.04	0.84 ± 0.04	0.87 ± 0.04	0.85 ± 0.04
	RF	0.78 ± 0.04	0.78 ± 0.04	0.81 ± 0.04	0.79 ± 0.04
	ResNet50 R	0.92 ± 0.03^{b}	0.92 ± 0.03^{b}	0.93 ± 0.03^{b}	0.92 ± 0.03^{b}
	Resnet50	0.90 ± 0.04^{b}	0.90 ± 0.04^{b}	0.93 ± 0.02^{b}	0.90 ± 0.03^{bc}
	MobileNetV2 R	0.91 ± 0.04^{b}	0.91 ± 0.04^{b}	0.92 ± 0.03^{b}	0.91 ± 0.03^{b,c}
	MobileNetV2	0.87 ± 0.04	0.87 ± 0.04	0.91 ± 0.03^{b}	0.88 ± 0.04^{c}

Note: For each performance metric, the performance values with the same superscript (a,b,c, and d) mean that there is no statistical difference among them ($p<0.05$).

Table S3: Tukey HSD test: accuracy comparisons across five OV grading classes.

Tukey's HSD Post-Hoc Test (5 classes) for accuracy:
Multiple Comparison of Means - Tukey HSD, FWER=0.05

group1	group2	meandiff	p-adj	lower	upper	reject
effc	effr	0.0344	0.3823	-0.0144	0.0833	False
effc	mnetc	-0.1494	0.0	-0.1983	-0.1006	True
effc	mnetr	-0.0789	0.0	-0.1277	-0.03	True
effc	rf	-0.1856	0.0	-0.2344	-0.1367	True
effc	rnetc	-0.0456	0.0876	-0.0944	0.0033	False
effc	rnetr	-0.0478	0.0604	-0.0966	0.0011	False
effc	svm	-0.1428	0.0	-0.1916	-0.0939	True
effr	mnetc	-0.1839	0.0	-0.2327	-0.135	True
effr	mnetr	-0.1133	0.0	-0.1622	-0.0645	True
effr	rf	-0.22	0.0	-0.2689	-0.1711	True
effr	rnetc	-0.08	0.0	-0.1289	-0.0311	True
effr	rnetr	-0.0822	0.0	-0.1311	-0.0334	True
effr	svm	-0.1772	0.0	-0.2261	-0.1284	True
mnetc	mnetr	0.0706	0.0004	0.0217	0.1194	True
mnetc	rf	-0.0361	0.32	-0.085	0.0127	False
mnetc	rnetc	0.1039	0.0	0.055	0.1527	True
mnetc	rnetr	0.1017	0.0	0.0528	0.1505	True
mnetc	svm	0.0067	0.9999	-0.0422	0.0555	False
mnetr	rf	-0.1067	0.0	-0.1555	-0.0578	True
mnetr	rnetc	0.0333	0.4266	-0.0155	0.0822	False
mnetr	rnetr	0.0311	0.5196	-0.0177	0.08	False
mnetr	svm	-0.0639	0.0021	-0.1127	-0.015	True
rf	rnetc	0.14	0.0	0.0911	0.1889	True
rf	rnetr	0.1378	0.0	0.0889	0.1866	True
rf	svm	0.0428	0.1346	-0.0061	0.0916	False
rnetc	rnetr	-0.0022	1.0	-0.0511	0.0466	False
rnetc	svm	-0.0972	0.0	-0.1461	-0.0484	True
rnetr	svm	-0.095	0.0	-0.1439	-0.0461	True

•

Table S4: Tukey HSD test: accuracy comparisons across two OV status classes.

Tukey's HSD Post-Hoc Test (2 classes) for accuracy:
Multiple Comparison of Means - Tukey HSD, FWER=0.05

group1	group2	meandiff	p-adj	lower	upper	reject
effc	effr	0.0022	1.0	-0.0259	0.0304	False
effc	mnetc	-0.0811	0.0	-0.1093	-0.053	True
effc	mnetr	-0.0439	0.0001	-0.072	-0.0157	True
effc	rf	-0.1717	0.0	-0.1998	-0.1435	True
effc	rnetc	-0.0528	0.0	-0.0809	-0.0246	True
effc	rnetr	-0.0294	0.0332	-0.0576	-0.0013	True
effc	svm	-0.1128	0.0	-0.1409	-0.0846	True
effr	mnetc	-0.0833	0.0	-0.1115	-0.0552	True
effr	mnetr	-0.0461	0.0	-0.0743	-0.018	True
effr	rf	-0.1739	0.0	-0.202	-0.1457	True
effr	rnetc	-0.055	0.0	-0.0831	-0.0269	True
effr	rnetr	-0.0317	0.0156	-0.0598	-0.0035	True
effr	svm	-0.115	0.0	-0.1431	-0.0869	True
mnetc	mnetr	0.0372	0.0018	0.0091	0.0654	True
mnetc	rf	-0.0906	0.0	-0.1187	-0.0624	True
mnetc	rnetc	0.0283	0.0472	0.0002	0.0565	True
mnetc	rnetr	0.0517	0.0	0.0235	0.0798	True
mnetc	svm	-0.0317	0.0156	-0.0598	-0.0035	True
mnetr	rf	-0.1278	0.0	-0.1559	-0.0996	True
mnetr	rnetc	-0.0089	0.9787	-0.037	0.0193	False
mnetr	rnetr	0.0144	0.7679	-0.0137	0.0426	False
mnetr	svm	-0.0689	0.0	-0.097	-0.0407	True
rf	rnetc	0.1189	0.0	0.0907	0.147	True
rf	rnetr	0.1422	0.0	0.1141	0.1704	True
rf	svm	0.0589	0.0	0.0307	0.087	True
rnetc	rnetr	0.0233	0.1859	-0.0048	0.0515	False
rnetc	svm	-0.06	0.0	-0.0881	-0.0319	True
rnetr	svm	-0.0833	0.0	-0.1115	-0.0552	True

Table S5: Tukey HSD test: recall comparisons across five OV grading classes.

Tukey's HSD Post-Hoc Test (5 classes) for recall:
Multiple Comparison of Means - Tukey HSD, FWER=0.05

group1	group2	meandiff	p-adj	lower	upper	reject
effc	effr	0.0344	0.3823	-0.0144	0.0833	False
effc	mnetc	-0.1494	0.0	-0.1983	-0.1006	True
effc	mnetr	-0.0789	0.0	-0.1277	-0.03	True
effc	rf	-0.1856	0.0	-0.2344	-0.1367	True
effc	rnetc	-0.0456	0.0876	-0.0944	0.0033	False
effc	rnetr	-0.0478	0.0604	-0.0966	0.0011	False
effc	svm	-0.1428	0.0	-0.1916	-0.0939	True
effr	mnetc	-0.1839	0.0	-0.2327	-0.135	True
effr	mnetr	-0.1133	0.0	-0.1622	-0.0645	True
effr	rf	-0.22	0.0	-0.2689	-0.1711	True
effr	rnetc	-0.08	0.0	-0.1289	-0.0311	True
effr	rnetr	-0.0822	0.0	-0.1311	-0.0334	True
effr	svm	-0.1772	0.0	-0.2261	-0.1284	True
mnetc	mnetr	0.0706	0.0004	0.0217	0.1194	True
mnetc	rf	-0.0361	0.32	-0.085	0.0127	False
mnetc	rnetc	0.1039	0.0	0.055	0.1527	True
mnetc	rnetr	0.1017	0.0	0.0528	0.1505	True
mnetc	svm	0.0067	0.9999	-0.0422	0.0555	False
mnetr	rf	-0.1067	0.0	-0.1555	-0.0578	True
mnetr	rnetc	0.0333	0.4266	-0.0155	0.0822	False
mnetr	rnetr	0.0311	0.5196	-0.0177	0.08	False
mnetr	svm	-0.0639	0.0021	-0.1127	-0.015	True
rf	rnetc	0.14	0.0	0.0911	0.1889	True
rf	rnetr	0.1378	0.0	0.0889	0.1866	True
rf	svm	0.0428	0.1346	-0.0061	0.0916	False
rnetc	rnetr	-0.0022	1.0	-0.0511	0.0466	False
rnetc	svm	-0.0972	0.0	-0.1461	-0.0484	True
rnetr	svm	-0.095	0.0	-0.1439	-0.0461	True

Table S6: Tukey HSD test: recall comparisons across two OV status classes.

Tukey's HSD Post-Hoc Test (2 classes) for recall:
Multiple Comparison of Means - Tukey HSD, FWER=0.05

group1	group2	meandiff	p-adj	lower	upper	reject
effc	effr	0.0022	1.0	-0.0259	0.0304	False
effc	mnetc	-0.0811	0.0	-0.1093	-0.053	True
effc	mnetr	-0.0439	0.0001	-0.072	-0.0157	True
effc	rf	-0.1717	0.0	-0.1998	-0.1435	True
effc	rnetc	-0.0528	0.0	-0.0809	-0.0246	True
effc	rnetr	-0.0294	0.0332	-0.0576	-0.0013	True
effc	svm	-0.1128	0.0	-0.1409	-0.0846	True
effr	mnetc	-0.0833	0.0	-0.1115	-0.0552	True
effr	mnetr	-0.0461	0.0	-0.0743	-0.018	True
effr	rf	-0.1739	0.0	-0.202	-0.1457	True
effr	rnetc	-0.055	0.0	-0.0831	-0.0269	True
effr	rnetr	-0.0317	0.0156	-0.0598	-0.0035	True
effr	svm	-0.115	0.0	-0.1431	-0.0869	True
mnetc	mnetr	0.0372	0.0018	0.0091	0.0654	True
mnetc	rf	-0.0906	0.0	-0.1187	-0.0624	True
mnetc	rnetc	0.0283	0.0472	0.0002	0.0565	True
mnetc	rnetr	0.0517	0.0	0.0235	0.0798	True
mnetc	svm	-0.0317	0.0156	-0.0598	-0.0035	True
mnetr	rf	-0.1278	0.0	-0.1559	-0.0996	True
mnetr	rnetc	-0.0089	0.9787	-0.037	0.0193	False
mnetr	rnetr	0.0144	0.7679	-0.0137	0.0426	False
mnetr	svm	-0.0689	0.0	-0.097	-0.0407	True
rf	rnetc	0.1189	0.0	0.0907	0.147	True
rf	rnetr	0.1422	0.0	0.1141	0.1704	True
rf	svm	0.0589	0.0	0.0307	0.087	True
rnetc	rnetr	0.0233	0.1859	-0.0048	0.0515	False
rnetc	svm	-0.06	0.0	-0.0881	-0.0319	True
rnetr	svm	-0.0833	0.0	-0.1115	-0.0552	True

Table S7: Tukey HSD test: precision comparisons across five OV grading classes.

Tukey's HSD Post-Hoc Test (5 classes) for precision:
Multiple Comparison of Means - Tukey HSD, FWER=0.05

group1	group2	meandiff	p-adj	lower	upper	reject
effc	effr	0.0362	0.4698	-0.0186	0.091	False
effc	mnetc	-0.1504	0.0	-0.2052	-0.0956	True
effc	mnetr	-0.0486	0.1236	-0.1034	0.0062	False
effc	rf	-0.178	0.0	-0.2328	-0.1232	True
effc	rnetc	-0.0464	0.165	-0.1012	0.0084	False
effc	rnetr	-0.0303	0.6946	-0.0851	0.0245	False
effc	svm	-0.1394	0.0	-0.1942	-0.0846	True
effr	mnetc	-0.1866	0.0	-0.2414	-0.1318	True
effr	mnetr	-0.0848	0.0001	-0.1396	-0.03	True
effr	rf	-0.2142	0.0	-0.269	-0.1594	True
effr	rnetc	-0.0826	0.0002	-0.1374	-0.0278	True
effr	rnetr	-0.0665	0.0062	-0.1213	-0.0117	True
effr	svm	-0.1756	0.0	-0.2304	-0.1209	True
mnetc	mnetr	0.1017	0.0	0.0469	0.1565	True
mnetc	rf	-0.0277	0.7819	-0.0825	0.0271	False
mnetc	rnetc	0.104	0.0	0.0492	0.1588	True
mnetc	rnetr	0.1201	0.0	0.0653	0.1749	True
mnetc	svm	0.0109	0.9987	-0.0439	0.0657	False
mnetr	rf	-0.1294	0.0	-0.1842	-0.0746	True
mnetr	rnetc	0.0022	1.0	-0.0526	0.057	False
mnetr	rnetr	0.0184	0.9702	-0.0364	0.0732	False
mnetr	svm	-0.0908	0.0	-0.1456	-0.036	True
rf	rnetc	0.1316	0.0	0.0768	0.1864	True
rf	rnetr	0.1478	0.0	0.093	0.2026	True
rf	svm	0.0386	0.3834	-0.0162	0.0934	False
rnetc	rnetr	0.0161	0.9858	-0.0387	0.0709	False
rnetc	svm	-0.093	0.0	-0.1478	-0.0382	True
rnetr	svm	-0.1092	0.0	-0.164	-0.0544	True

Table S8: Tukey HSD test: F1-score comparisons across five OV grading classes.

```
Tukey's HSD Post-Hoc Test (5 classes) for f1_score:
Multiple Comparison of Means - Tukey HSD, FWER=0.05
====================================================
group1 group2 meandiff p-adj   lower   upper  reject
----------------------------------------------------
  effc   effr   0.0353 0.3954 -0.0153  0.0858  False
  effc  mnetc  -0.1609    0.0 -0.2114 -0.1104   True
  effc  mnetr   -0.074 0.0003 -0.1245 -0.0235   True
  effc     rf  -0.1856    0.0 -0.2361  -0.135   True
  effc  rnetc  -0.0565 0.0167  -0.107  -0.006   True
  effc  rnetr  -0.0434 0.1515 -0.0939  0.0071  False
  effc    svm  -0.1464    0.0  -0.197 -0.0959   True
  effr  mnetc  -0.1962    0.0 -0.2467 -0.1456   True
  effr  mnetr  -0.1093    0.0 -0.1598 -0.0588   True
  effr     rf  -0.2208    0.0 -0.2714 -0.1703   True
  effr  rnetc  -0.0918    0.0 -0.1423 -0.0412   True
  effr  rnetr  -0.0787 0.0001 -0.1292 -0.0281   True
  effr    svm  -0.1817    0.0 -0.2322 -0.1312   True
 mnetc  mnetr   0.0869    0.0  0.0364  0.1374   True
 mnetc     rf  -0.0247 0.8099 -0.0752  0.0258  False
 mnetc  rnetc   0.1044    0.0  0.0539  0.1549   True
 mnetc  rnetr   0.1175    0.0   0.067   0.168   True
 mnetc    svm   0.0145  0.988 -0.0361   0.065  False
 mnetr     rf  -0.1116    0.0 -0.1621  -0.061   True
 mnetr  rnetc   0.0175 0.9641  -0.033   0.068  False
 mnetr  rnetr   0.0306 0.5845 -0.0199  0.0811  False
 mnetr    svm  -0.0724 0.0005 -0.1229 -0.0219   True
    rf  rnetc   0.1291    0.0  0.0786  0.1796   True
    rf  rnetr   0.1422    0.0  0.0916  0.1927   True
    rf    svm   0.0391 0.2614 -0.0114  0.0897  False
 rnetc  rnetr   0.0131 0.9934 -0.0374  0.0636  False
 rnetc    svm  -0.0899    0.0 -0.1405 -0.0394   True
 rnetr    svm   -0.103    0.0 -0.1536 -0.0525   True
----------------------------------------------------
```

Table S9: Tukey HSD test: F1-score comparisons across two OV status classes.

Tukey's HSD Post-Hoc Test (2 classes) for f1_score:
Multiple Comparison of Means - Tukey HSD, FWER=0.05

group1	group2	meandiff	p-adj	lower	upper	reject
effc	effr	0.0036	0.9999	-0.0227	0.0298	False
effc	mnetc	-0.0698	0.0	-0.096	-0.0436	True
effc	mnetr	-0.0402	0.0001	-0.0664	-0.014	True
effc	rf	-0.1613	0.0	-0.1875	-0.1351	True
effc	rnetc	-0.044	0.0	-0.0702	-0.0177	True
effc	rnetr	-0.0289	0.0193	-0.0552	-0.0027	True
effc	svm	-0.1006	0.0	-0.1268	-0.0744	True
effr	mnetc	-0.0734	0.0	-0.0996	-0.0472	True
effr	mnetr	-0.0438	0.0	-0.07	-0.0175	True
effr	rf	-0.1648	0.0	-0.1911	-0.1386	True
effr	rnetc	-0.0475	0.0	-0.0738	-0.0213	True
effr	rnetr	-0.0325	0.0047	-0.0587	-0.0063	True
effr	svm	-0.1042	0.0	-0.1304	-0.0779	True
mnetc	mnetr	0.0296	0.0148	0.0034	0.0559	True
mnetc	rf	-0.0915	0.0	-0.1177	-0.0652	True
mnetc	rnetc	0.0259	0.0564	-0.0004	0.0521	False
mnetc	rnetr	0.0409	0.0001	0.0147	0.0671	True
mnetc	svm	-0.0308	0.0095	-0.057	-0.0045	True
mnetr	rf	-0.1211	0.0	-0.1473	-0.0949	True
mnetr	rnetc	-0.0038	0.9999	-0.03	0.0224	False
mnetr	rnetr	0.0113	0.8931	-0.015	0.0375	False
mnetr	svm	-0.0604	0.0	-0.0866	-0.0342	True
rf	rnetc	0.1173	0.0	0.0911	0.1435	True
rf	rnetr	0.1324	0.0	0.1061	0.1586	True
rf	svm	0.0607	0.0	0.0345	0.0869	True
rnetc	rnetr	0.015	0.6517	-0.0112	0.0413	False
rnetc	svm	-0.0566	0.0	-0.0829	-0.0304	True
rnetr	svm	-0.0717	0.0	-0.0979	-0.0454	True
